# Molecular simulations reveal intricate coupling between agonist-bound β-adrenergic receptors and G protein

**DOI:** 10.1016/j.isci.2024.111741

**Published:** 2025-01-02

**Authors:** Yanxiao Han, John R.D. Dawson, Kevin R. DeMarco, Kyle C. Rouen, Khoa Ngo, Slava Bekker, Vladimir Yarov-Yarovoy, Colleen E. Clancy, Yang K. Xiang, Surl-Hee Ahn, Igor Vorobyov

**Affiliations:** 1 Department of Physiology and Membrane Biology, University of California, Davis, Davis, CA 95616, USA; 2 Biophysics Graduate Group, University of California, Davis, Davis, CA 95616, USA; 3 American River College, Sacramento, CA 95841, USA; 4 Department of Anesthesiology and Pain Medicine, University of California, Davis, Davis, CA 95616, USA; 5 Center for Precision Medicine and Data Science, University of California, Davis, Davis, CA 95616, USA; 6 Department of Pharmacology, University of California, Davis, Davis, CA 95616, USA; 7 VA Northern California Health Care System, Mather, CA 95655, USA; 8 Department of Chemical Engineering, University of California, Davis, Davis, CA 95616, USA

**Keywords:** Protein structure aspects, Computational bioinformatics

## Abstract

G protein-coupled receptors (GPCRs) and G proteins transmit signals from hormones and neurotransmitters across cell membranes, initiating downstream signaling and modulating cellular behavior. Using advanced computer modeling and simulation, we identified atomistic-level structural, dynamic, and energetic mechanisms of norepinephrine (NE) and stimulatory G protein (G_s_) interactions with β-adrenergic receptors (βARs), crucial GPCRs for heart function regulation and major drug targets. Our analysis revealed distinct binding behaviors of NE within β_1_AR and β_2_AR despite identical orthosteric binding pockets. β_2_AR had an additional binding site, explaining variations in NE binding affinities. Simulations showed significant differences in NE dissociation pathways and receptor interactions with the G_s_. β_1_AR binds G_s_ more strongly, while β_2_AR induces greater conformational changes in the α subunit of G_s_. Furthermore, GTP and GDP binding to G_s_ may disrupt coupling between NE and βAR, as well as between βAR and G_s_. These findings may aid in designing precise βAR-targeted drugs.

## Highlights

• Additional binding site is present in β_2_AR but not in β_1_AR
• Distinct NE dissociation pathways exist for β_1_AR and β_2_AR
• β_1_AR binds G_s_ more strongly than β_2_AR
• GTP/GDP binding may disrupt NE-βAR and βAR-G_s_ coupling

## Graphical abstract

**Figure:**
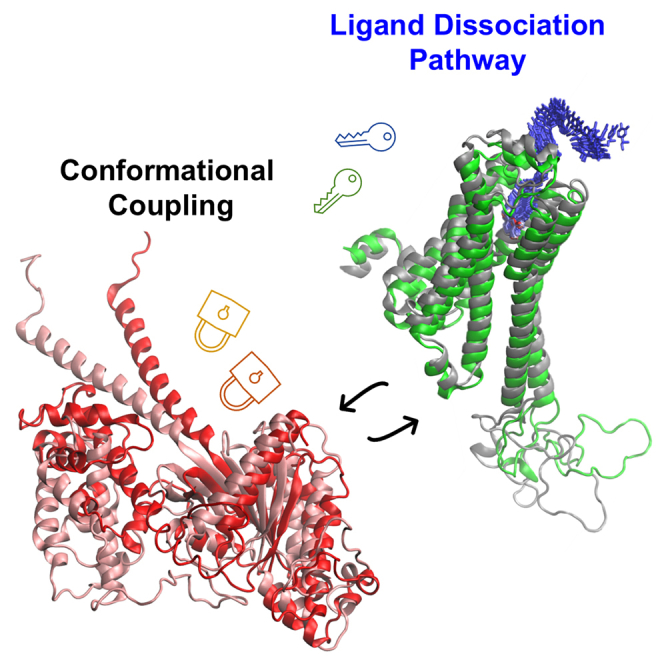

## Introduction

β-adrenergic receptors (βARs) are a vital class of G protein-coupled receptors (GPCRs) that respond to catecholamines produced by the body and medications used to treat cardiac diseases.^1^^,^^2^^,^^3^^,^^4^ There are three βAR subtypes in the nonfailing human heart (β_1_ accounts for 75%–80%, β_2_ 15%–18%, and β_3_ 2%–3%),^5^ which regulate the cardiac rate and contractility by responding to endogenous ligands, norepinephrine (NE) and epinephrine (Epi).^6^^,^^7^ However, the ratio of β_1_ and β_2_ subtypes in the failing human heart becomes approximately equal.^8^ β_1_ARs primarily couple to the stimulatory G protein (G_s_), leading to the synthesis of cyclic adenosine 3′,5′-monophosphate (cAMP) by the enzyme adenylyl cyclase (AC). The activation of the G_s_ pathway increases heart rate and myocardial contractility. In contrast, β_2_ARs are pleiotropic receptors that can couple to both G_s_ and the inhibitory G protein, G_i_.^9^ Activation of the β_2_AR/G_i_ pathway inhibits cAMP production, which opposes the effect of G_s_ activation.^10^

GPCR is one of the most successful therapeutic protein target families. These membrane proteins often translate outside extracellular molecular signals in the form of endogenous hormones, neurotransmitters, drug molecules, and peptides into intracellular signaling responses by interacting with G proteins. Comprehensive review articles about GPCRs can be found here.^11^^,^^12^^,^^13^ G proteins, or guanine nucleotide-binding proteins, consist of Gα, Gβ, and Gγ subunits. They are categorized based on their Gα subunits, which include Gα_s_, Gα_i_, Gα_q/11_, and Gα_12/13_.^14^ G proteins exist in inactive or active states depending on whether the nucleotide bound to Gα is guanosine diphosphate (GDP) or guanosine triphosphate (GTP). Specifically, GDP-bound Gα forms an inactive trimeric complex with Gβγ, whereas GTP-bound Gα exists in an active state dissociated from both receptor and Gβγ subunits.^15^ Conformational dynamics of a Gα subunit is tightly regulated by nucleotide binding.^16^ Several structures have revealed that the nucleotide-binding pocket is located between the Ras-like GTPase domain (RD) and the α-helical domain (AHD) of Gα.^17^

Previous studies used extensive molecular dynamics simulations to discover ligand-specific conformations within β_2_AR^18^^,^^19^ and other GPCRs.^20^ Those studies revealed that ligands of varying efficacies, such as inverse agonists, neutral antagonists, or agonists, influence the receptor’s free-energy landscape, which alters the conformational equilibrium, promoting active or inactive states.^18^^,^^19^^,^^20^ GPCRs have multiple ligand binding sites, the orthosteric binding pocket (OBP), and a generally less conserved allosteric secondary binding pocket (SBP) separated from OBP.^21^^,^^22^ Endogenous ligands are known to engage with the OBP situated within the intrahelical region of the receptor,^22^ while SBPs in numerous receptors are primarily located within the extracellular vestibule, such as Cmpd-15PA and AS408 binding to β_2_AR (PDB codes: 5X7D^23^ and 6OBA^24^), and other compounds binding to different GPCRs.^25^^,^^26^^,^^27^^,^^28^^,^^29^ It was also found that ligands can bind to an extended OBP, such as the compound MK-6892 in the hydroxycarboxylic acid receptor 2^30^ and aripiprazole in the 5-hydroxytryptamine (serotonin) 2A receptor.^31^ Those SBPs are primarily situated above the OBP, toward the extracellular region. A deeper allosteric site toward the intracellular region was discovered through computational site identification methods on an intermediate conformation of β_2_AR.^32^ Selvam et al. simulated unbinding of the β_1_AR-selective drug (esmolol) and β_2_AR-selective drug (ICI-118551) from both receptors to the extracellular environment and found distinct amino acid residues and interactions, which drive the ligand selectivity.^33^ Simulations of two inverse agonists, cyanopindolol and carazolol, co-crystallized with β_1_AR and β_2_AR indicate the presence of secondary binding sites in the extracellular loops (ECLs) 2 and 3 and transmembrane helix (TM)7.^34^ Using simulations, researchers also explored the dynamics of Epi in the OBP of β_2_AR, revealing the existence of two distinct stable states for the Epi-β_2_AR complex: a global energy minimum and a meta-stable state separated by an energy barrier.^35^ Xu et al. recently discovered that both β_1_AR and β_2_AR share identical OBP residues for NE and Epi.^36^ They also found that NE exhibits approximately 10-fold selectivity for β_1_AR over β_2_AR, whereas Epi is less selective, which they thought was due to the different binding (entrance) pathway of NE in the two receptor subtypes.^36^ They further found that conformationally constrained Epi gains selectivity for β_2_AR over β_1_AR, which they thought might be due to allosteric effects of surrounding residues, especially the ECLs forming the vestibule.^37^ We propose that ligand selectivity can also be influenced by an additional binding site resulting from the allosteric effects of residues in β_2_AR, which are absent in β_1_AR.

The conformational dynamics of G_s_α associated with nucleotide exchange were studied extensively. Rasmussen et al. showed that, in the ternary complex of ligand-β_2_AR-G_s_, G_s_ binding increased the agonist binding affinity about 100-fold compared with β_2_AR alone; agonist binding promoted interactions of β_2_AR with GDP-bound G_s_ heterotrimer leading to the exchange of GDP for GTP.^38^ Su et al. found that β_1_AR induced a tilting of the α5-helix of G_s_α, which deformed the GDP/GTP-binding pocket and accelerated GDP release; they also proposed the possibility of a subsequent weak GTP binding site on the open G_s_α.^39^ Dror et al. studied the structural basis for GDP/GTP exchange in G_s_ proteins by combining long-timescale molecular dynamics (MD) simulations with experimental validations. Through simulations, they found that an internal structural rearrangement of the G_s_α RD was needed to weaken its nucleotide affinity.^40^ The active structure of the β_2_AR stabilized only by the last 14 residues of the G_s_α α5 helix, crystallized by Liu et al., showed an intermediate state between the GDP-bound G_s_ and the formation of the GDP-free β_2_AR-G_s_ complex.^41^ Using MD simulations, Batebi et al. revealed the structural rearrangement of GDP-bound G_s_ during its association with β_2_AR and observed the long-range allosteric effects of G_s_ triggering GDP release using MD simulations.^42^ Alhadeff et al. explored the free-energy landscape of β_2_AR activation using coarse-grained modeling combining multiple receptor and G_s_ protein conformational states. They found that the transition of the G_s_ protein from the closed to the open state reduced the binding affinity of GDP but had little effect on the affinity of GTP.^43^ Bai et al. coupled β_2_AR-G_s_α conformational changes with GDP release to generate a free-energy map, identifying a pathway for GDP release. They found that GDP could be released to the bulk solvent after G_s_ was half open or remained in the pocket until the stable β_2_AR-G_s_ nucleotide-free complex was formed. The potential key residues on α5 affecting the G protein coupling and GDP release were also validated by site-directed mutagenesis.^44^ Simulation work also revealed that GDP release from the open conformation of G_s_α requires allosteric signaling from the agonist (BI-167107)-bound β_2_AR.^45^ The binding of GDP to the β_2_AR triggers allosteric effects that lower the energy needed for GDP release, involving the opening and displacement of specific helices in the G_s_α.^46^

Recently, Ahn et al. studied the dissociation of G_s_α from β_2_AR and found that GTP binding and GTP-induced dissociation of G_s_α from β_2_AR and Gβγ were much faster than the closing of AHD by combining data from hydrogen-deuterium exchange mass spectrometry, tryptophan-induced fluorescence quenching, and metadynamics simulations.^47^ While fundamental difference exists between β_1_AR and β_2_AR in terms of ligand binding rates and ligand binding pathway,^36^^,^^37^ it remains unclear whether similar nucleotide-induced G_s_α dissociation can be found in β_1_AR. We hypothesize that the dissociation dynamics of G_s_α may vary between β_1_AR and β_2_AR, contributing to their distinct downstream signaling effects. We also tested the effect of nucleotide (GTP or GDP) binding, which may cause different conformational dynamics of G_s_α when it interacts with β_1_AR and β_2_AR. Assessing the ligand dynamics in βARs, ligand modulation of βARs coupling to G_s_ protein, and the effect of guanine nucleotides in the coupling between G protein and βARs is essential for understanding the physiological functions of GPCRs and G proteins and their downstream signaling pathways in cardiac function modulations in health and disease. This will offer valuable insights into the distinct characteristics and functions of these receptors and their roles in orchestrating cellular signaling pathways that regulate cardiac function.

**Figure 1:**
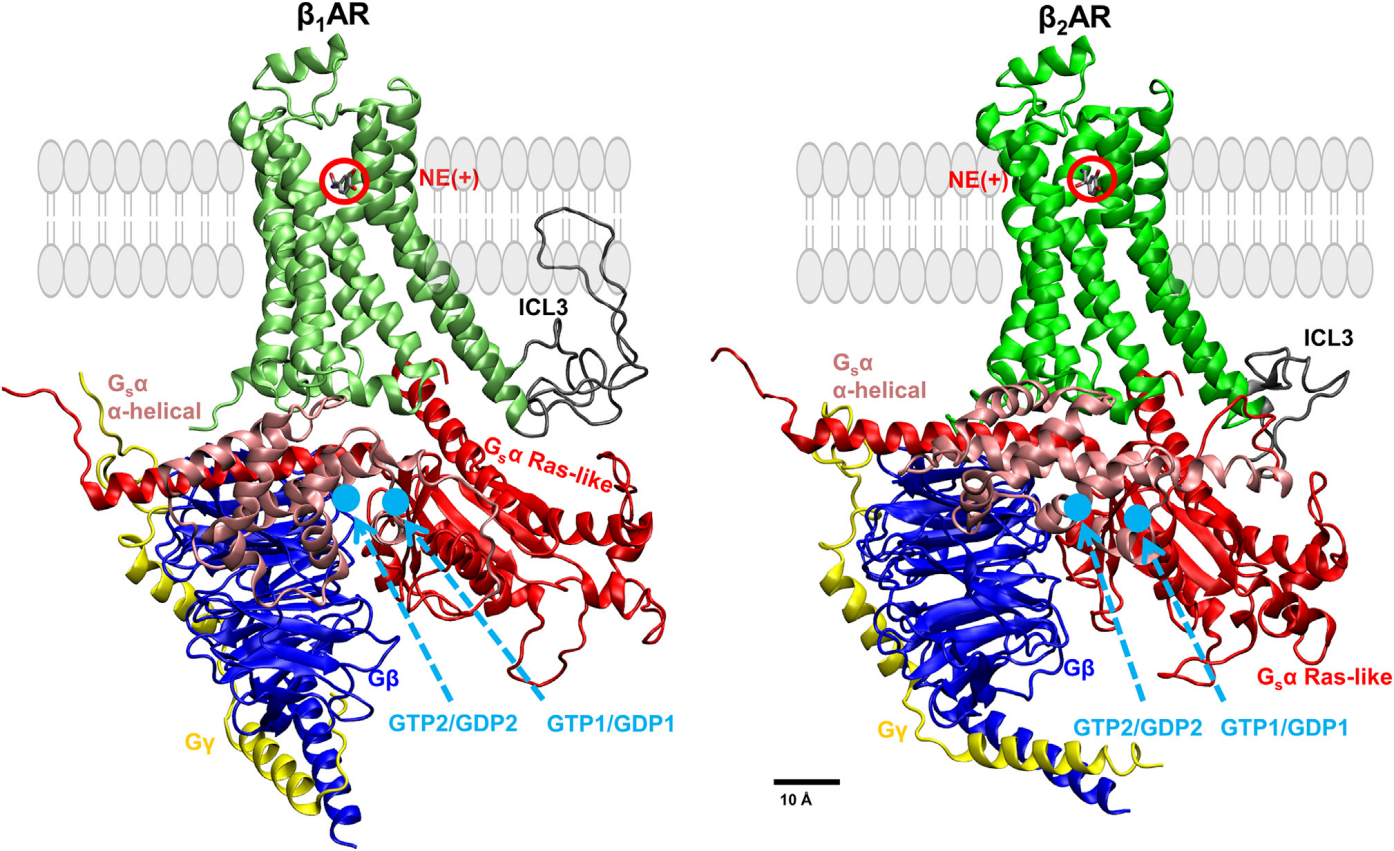
Active state human β_1_AR and β_2_AR coupled with G_s_ protein Different subunits and loops are illustrated by different colors. Lime, β_1_AR; green, β_2_AR; gray, βAR intracellular loop 3 (ICL3); pink, G_s_α α-helical domain; red, G_s_α Ras-like domain; blue, Gβ; and yellow, Gγ. NE(+) is circled out in red. GTP/GDP docking positions 1 and 2 are marked in light blue. Approximate lipid membrane position is shown by light-gray cartoons.

In this study, we explored the differences of cationic NE, NE(+), binding dynamics; its complete dissociation from β_1_AR and β_2_AR; the effects of guanine nucleotides (GTP and GDP) binding to the G_s_α conformational changes; and G_s_α partial dissociation from both β_1_AR and β_2_AR. Our MD simulation starting points were based on experimental PDB structures, as shown in Figure 1. We conducted extensive, unbiased MD simulations spanning multiple microseconds alongside shorter Gaussian accelerated MD (GaMD) simulations and multiple MD simulation runs using the weighted ensemble (WE) method. GaMD^48^ and WE^49^ differ from methods like funnel metadynamics.^50^ GaMD offers the advantage of not requiring any collective variables, while WE is a statistically unbiased method. A comprehensive review of these and other enhanced sampling methods is available here.^51^ These investigations aimed to elucidate the molecular interactions within systems comprising NE(+) and either the β_1_AR or β_2_AR, as well as G_s_ coupled with nucleotides (GTP or GDP). We introduced the term “conformational coupling,” representing the structural fitness between two molecular system components. Employing a machine learning-based method, we assessed the fitness of two adjacent components in our systems and examined the effects of G_s_ and nucleotides on receptors and ligand binding. We identified potential conformational coupling pairs, resembling “key-lock” pairs, between ligands and receptors and between receptors and G protein.

## Results

Two structural models of β_1_AR and β_2_AR in their active state were prepared and then coupled with the stimulatory G_s_, forming β_1_AR-G_s_ and β_2_AR-G_s_ systems, respectively. The snapshots of the initial β_1_AR-G_s_ and β_2_AR-G_s_ systems are shown in Figure 1. The known nucleotide-binding pocket is located between the RD and AHD of a closed G_s_α.^17^ There is no structure available for the nucleotide-bound G_s_α in an open state. We then conducted molecular docking to position the nucleotide in two distinct locations: one interacting with the AHD (labeled as 1), while the other interacting with the RD of G_s_α (labeled as 2). The magnesium ion was absent due to the lack of nucleotide-bound open G_s_α structure and in line with previous simulations of the related systems.^40^ However, during MD simulations, an appropriate number of Na^+^ and Cl^−^ ions were introduced to neutralize the systems and sample physiological ion concentration properly. We found that the P loop near RD is a common binding site for GTP and GDP. The top binding poses were selected for each docking position, forming four β_1_AR systems (β_1_AR-G_s_-GTP1, β_1_AR-G_s_-GTP2, β_1_AR-G_s_-GDP1, and β_1_AR-G_s_-GDP2) and four β_2_AR systems (β_2_AR-G_s_-GTP1, β_2_AR-G_s_-GTP2, β_2_AR-G_s_-GDP1, and β_2_AR-G_s_-GDP2). NE(+), bound at the OBP of βAR, was present in each system. The numbering codes assigned to GTP/GDP binding positions denote their initial docking locations, such as GTP1, which indicates its initial placement near AHD. Each system was embedded in a lipid bilayer hydrated by 0.15 M NaCl, corresponding to physiological conditions in the extracellular medium, and equilibrated for 90 ns using gradually reducing restraints in the 1^st^ 40 ns of these simulations. Then, much longer production runs followed. We performed a 2.5 μs Anton 2 unrestrained MD simulation for each system and a 300 ns GaMD run. For the NE-βAR systems, we performed additional simulations using WE to sample the full dissociation of NE from βARs. Based on the MD simulation trajectories, we first checked the dominant and secondary NE(+) binding poses in the GTP/GDP-bound βAR-G_s_ systems and analyzed the role of GTP/GDP coupling to G_s_ in stabilizing/destabilizing the NE(+) binding. Then, we assessed the conformational changes in the G_s_α upon coupling with the guanine nucleotides. The interaction between intracellular loop 3 (ICL3) of βAR and G_s_α α5 helix was also analyzed. Multiple structural parameters were analyzed to find the molecular determinants of G_s_α conformational changes, including the opening/closing of G_s_α and the distance between two G_s_α domains. We then analyzed the correlation between any of those structural parameters, followed by the analysis of conformational coupling between NE(+) and βAR and between βAR and G_s_. Key amino acid residues were identified in those analyses, and one-letter residue names followed by their number as well as Ballesteros-Weinstein numbering^52^ for βARs in parentheses (when known) will be shown. We also performed a posteriori implicit-solvent molecular mechanics Poisson-Boltzmann surface area (MM-PBSA) calculations^53^ to estimate relative trends in βAR binding to NE and G_s_, respectively.

### Distinct NE binding behaviors were found for β_1_AR and β_2_AR with key amino acid residues and transmembrane helices identified

**Figure 2:**
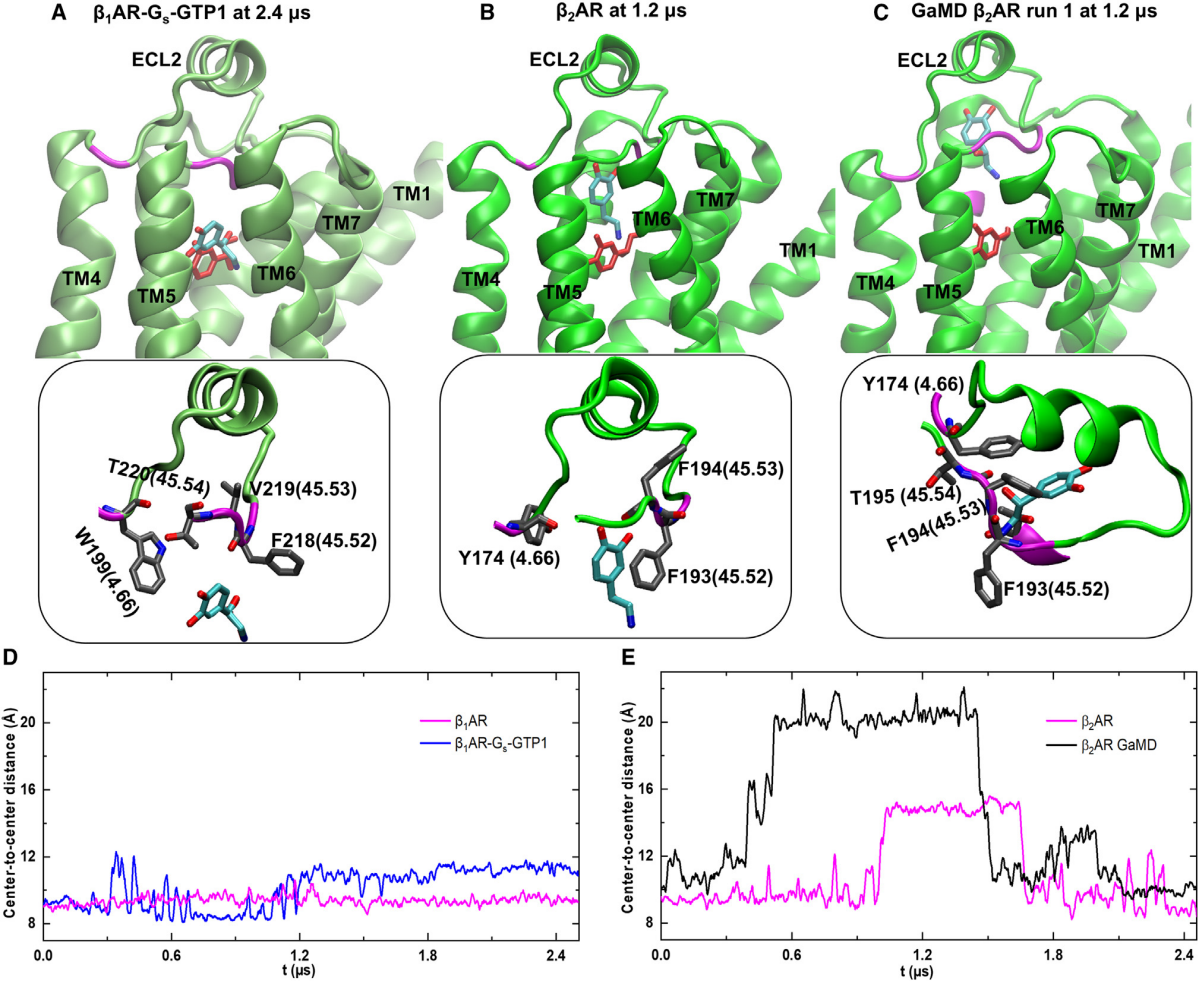
NE(+) partial dissociation during MD simulation from β_1_AR- and β_2_AR-containing systems (A) NE(+) binding pose captured in the β_1_AR-G_s_-GTP1 system. (B) NE(+) binding pose captured in the β_2_AR system. (C) the most dislocated NE(+) pose captured in β_2_AR GaMD simulation. The red molecule indicates the initial position of NE(+), while the cyan molecule indicates partially dissociated NE(+). Receptors are shown by green cartoons. The insets delineate the particular amino acid residue interactions with NE(+), corresponding to their respective main figures depicted above. The residues were indicated by their names, residue numbers, and Ballesteros-Weinstein numbers. (D) Time series of center-to-center distances (CCD) between NE(+) and β_1_AR systems. (E) Time series of center-to-center distances between NE(+) and β_2_AR systems. For the CCD, the geometric centers of NE(+) and βAR (excluding ICL3 and C-terminal residues) were used.

Our multi-microsecond Anton 2 MD and GaMD simulations, targeting the NE(+) partial dissociation, revealed a secondary binding site of NE(+) after dislocation from the OBP in β_2_AR. A series of snapshots (labeled as A through C) display the NE(+) poses that were the most dislocated in both β_1_AR and β_2_AR before NE(+) full dissociation (Figures 2 and S1). The initial NE(+) poses and interactions with β_1_AR and β_2_AR are shown in Figures S1C and S1D. These poses were obtained using either Anton 2 MD or GaMD simulations. The center-to-center distance (CCD) between the NE(+) and βAR systems covers a variety of scenarios, including those for systems with and without G_s_, as well as those for systems with or without GTP/GDP binding (Figures 2D, 2E, and S2). For the CCD, the geometric centers of NE(+) and βAR (excluding ICL3 and C-terminal residues) were used.

The cyan NE(+) molecule in Figure 2A depicted the highest level of dislocation in β_1_AR-G_s_-GTP1 among all the β_1_AR systems, corresponding to a CCD of ∼12.5 Å at approximately 2.4 μs (Figure 2D). The CCD plot can be divided into four distinct regions: an early transient partial dissociation event centered around 0.35 μs, a second wave of smaller transient partial dissociation events centered around 0.6 μs, a rebinding event centered around 0.9 μs, and a sustained partial dissociation event after 1.2 μs. To capture the minor dissociation of NE(+) in β_1_AR, we selected representative poses at specific time points: 0.35 (pose 1), 0.6 (pose 2), 0.9 (pose 3), and 1.5 μs (pose 4), as shown in Figure S1E. The amino acid residues interacting with NE(+) at various time points, color-coded to match the corresponding NE(+) pose, were shown in Figure S1F. Poses 1, 3, and 4 share the same amino acid residue contacts with β_1_AR in the β_1_AR-G_s_-GTP1 system, while pose 2 has an additional contact with β_1_AR residue V142(3.36). Compared to the initial binding pose, poses 1 through 4 form new NE(+) contacts with β_1_AR residues T220(5.34), V142(3.36), and W337(6.48) but lose contact with S232(5.46). The Anton 2 MD simulation captured the most dislocated NE(+) in the β_2_AR system before NE(+) full dissociation (Figure 2B). This NE(+) pose corresponds to the simulation time between 1.0 and 1.7 μs in the magenta plot (Figure 2E), with a CCD of approximately 15 Å. The results of NE(+)-β_2_AR and NE(+)-β_2_AR-G_s_ simulations were reported in our recent paper,^54^ where we found that the most dislocated NE(+) pose captured in the β_2_AR-G_s_ system was distinct from the one captured in the β_2_AR-only system. This suggests that the binding of G_s_ significantly impacts the binding modes of NE(+),^54^ which was also corroborated by experiments regarding the binding affinity enhancement of NE in the β_2_AR-G_s_ system.^38^ In the GaMD run for β_2_AR, an additional binding site for NE(+) with CCD of about 20 Å was sampled (Figure 2C). The two distinct binding sites of NE(+) during dissociation from β_2_AR with CCDs of 15 Å and 20 Å (Figures 2B and 2C) are significant because they differ from the NE(+) sites captured in previous metadynamics simulations of NE(+) association (with a CCD of 12 Å).^36^ This suggests that the NE(+) dissociation pathway can differ from its association (binding) pathway. Our interpretation is that the secondary binding site of NE(+) in β_2_AR acts as a bottleneck, obstructing both the entry and exit of NE(+). This phenomenon could explain the experimentally observed lower rate constants for both association and dissociation of NE in β_2_AR compared to those observed in β_1_AR.^36^

**Figure 3:**
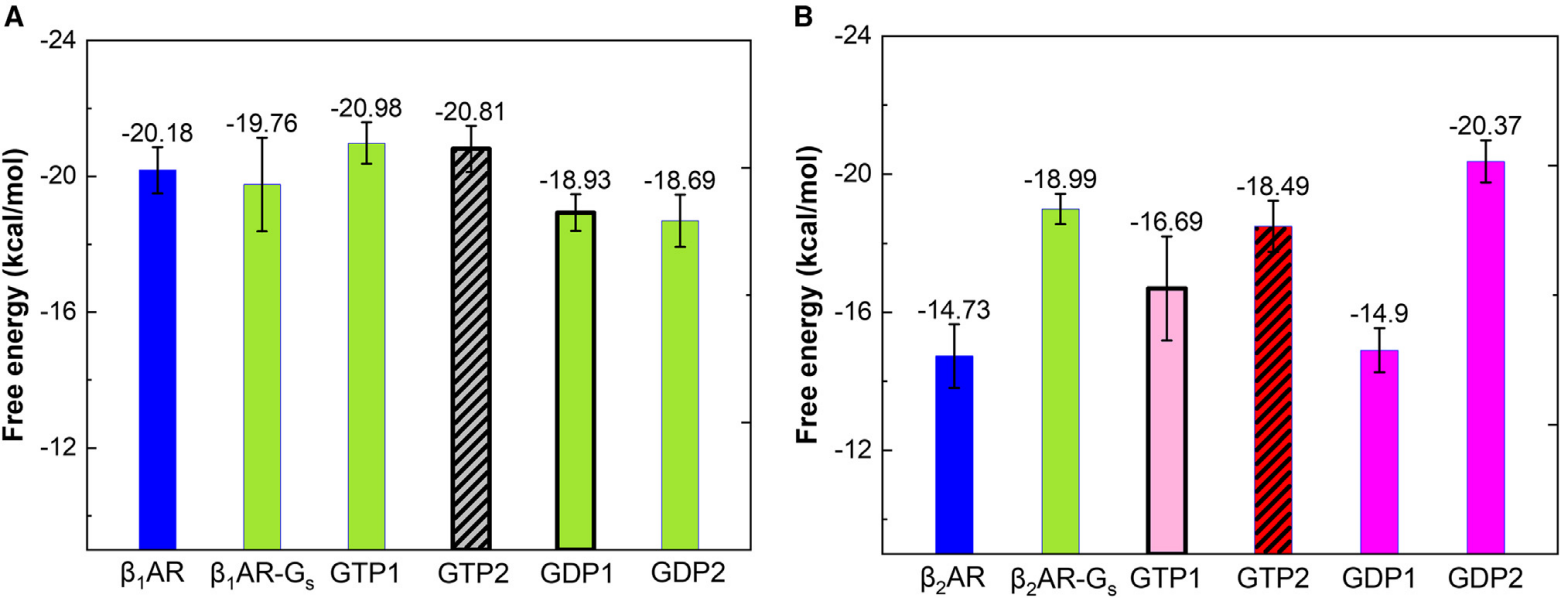
MM-PBSA free energies of binding (in kcal/mol) between NE(+) and βARs, based on the last 1 μs of Anton 2 MD simulation trajectories (A) is for β_1_AR systems and (B) is for β_2_AR systems with and without G_s_ and GDP/GTP bound at sites 1 and 2. Standard errors of the mean (SEMs) shown as error bars were computed using block averages. Color codes for the dominant conformation of G_s_ during those simulations: green, open; gray with stripes, semi-open to open; pink, flipped up; red with stripes, open to closed; magenta, semi-closed; black bold frames of the boxes indicate that GTP/GDP was bound during the last 1 μs of the simulation.

To determine the free energy of binding between NE(+) and βARs, we employed the MM-PBSA method (Figure 3 and Table S1). Our findings indicate that NE(+) binds stronger to β_1_AR (−20.18 ± 0.68 kcal/mol) than to β_2_AR (−14.73 ± 0.92 kcal/mol),^54^ which is consistent with the results of experimental binding assays reported previously.^36^ However, it is unclear whether G_s_ binding can stabilize NE(+) binding to β_1_AR, as observed in the case of β_2_AR because the β_1_AR-G_s_ complex exhibits an NE(+) binding energy (−19.76 ± 1.38 kcal/mol) that is similar to that of the β_1_AR system.

Tables S3–S5 list the residues contributing to the NE(+) binding in its most dislocated poses. In β_2_AR, the TM4 and ECL2 residues Y174(4.66), F194(45.53), and F193(45.52) form an additional binding pocket through allosteric effects that can trap NE(+) during its dislocation (Figures 2B and 2C). Y174(4.66) with both the aromatic ring and the hydroxyl group is crucial in facilitating the interaction with NE(+), which has the same functional groups. NE(+) can interact with Y174(4.66) in a one-to-one interaction mode via hydrophobic and hydrogen bonding interactions. In contrast, no tyrosine residue is found around the most dislocated NE(+) in β_1_AR. However, we found residues W199(4.66) and T220(45.54) in β_1_AR as shown in Figure 2A, which can be the equivalents of Y174(4.66) in β_2_AR, with W199(4.66) having an aromatic ring and T220(45.54) having a hydroxyl group. NE(+) can interact with these two amino acid residues in a one-to-two mode, potentially resulting in a less specific and less stable interaction. A similar effect was found in the recent experiments where the conformationally free Epi showed low selectivity for β_2_AR, while the conformationally constrained Epi gained enhanced affinity for β_2_AR.^37^

ECL2 residue F(45.52) is conserved in both β_1_AR and β_2_AR. Mutagenesis studies revealed that four residues surrounding F(45.52) but not directly interacting with Epi are important in stabilizing its binding in βAR.^37^ Interestingly, TM4 residues Y174(4.66) in β_2_AR and W199(4.66) in β_1_AR recognized as crucial for NE(+) binding by our simulations were also claimed as key amino acid residues in regulating Epi binding in the experimental mutagenesis study.^37^ This indicates that Y174 and W199 could also influence NE(+) binding due to the structural similarity between NE and Epi.

In addition, the root-mean-square deviations (RMSDs) of the coordinates of TM1 to TM7 were calculated for the cases exhibiting NE(+) dislocation, namely β_1_AR-G_s_-GTP1, β_2_AR-only, β_2_AR-G_s_, and β_2_AR-G_s_-GTP1 systems (Table S6). The numbering of the helices can be referred to in Figure S1. Then, a correlation analysis was performed between the RMSD of NE(+) and the individual helix in βAR over the MD simulation time to interpret the correlation of NE(+) movements and that of the individual helix. For β_1_AR systems, NE(+) partial dissociation was only observed in β_1_AR-G_s_-GTP1, where the RMSDs of TM3 and TM4 showed the strongest correlations with that of NE(+)—with Pearson correlation coefficients *r* of 0.58 and 0.51, respectively (see Table S6). For β_2_AR, a positive correlation was observed between NE(+) RMSD and those of TM3 (*r* = 0.57) and TM4 (*r* = 0.87). Figure S3 shows the conformation of helices TM3 (pink) and TM4 (black) when NE(+) in the β_2_AR system shows the highest level of partial dissociation at ∼1.2 μs. In the presence of G_s_, positive correlations were discovered between NE(+) RMSD and those of TM1, TM3, and TM6. For the β_2_AR-G_s_-GTP1 system, the correlation between NE(+) RMSD and RMSDs of helices was very weak. Our results suggest that TM3 and TM4 of β_2_AR are the major contributors to NE(+) partial dissociation, but the presence of G_s_ and GTP could modify the correlations between the helices and NE(+).

**Figure 4:**
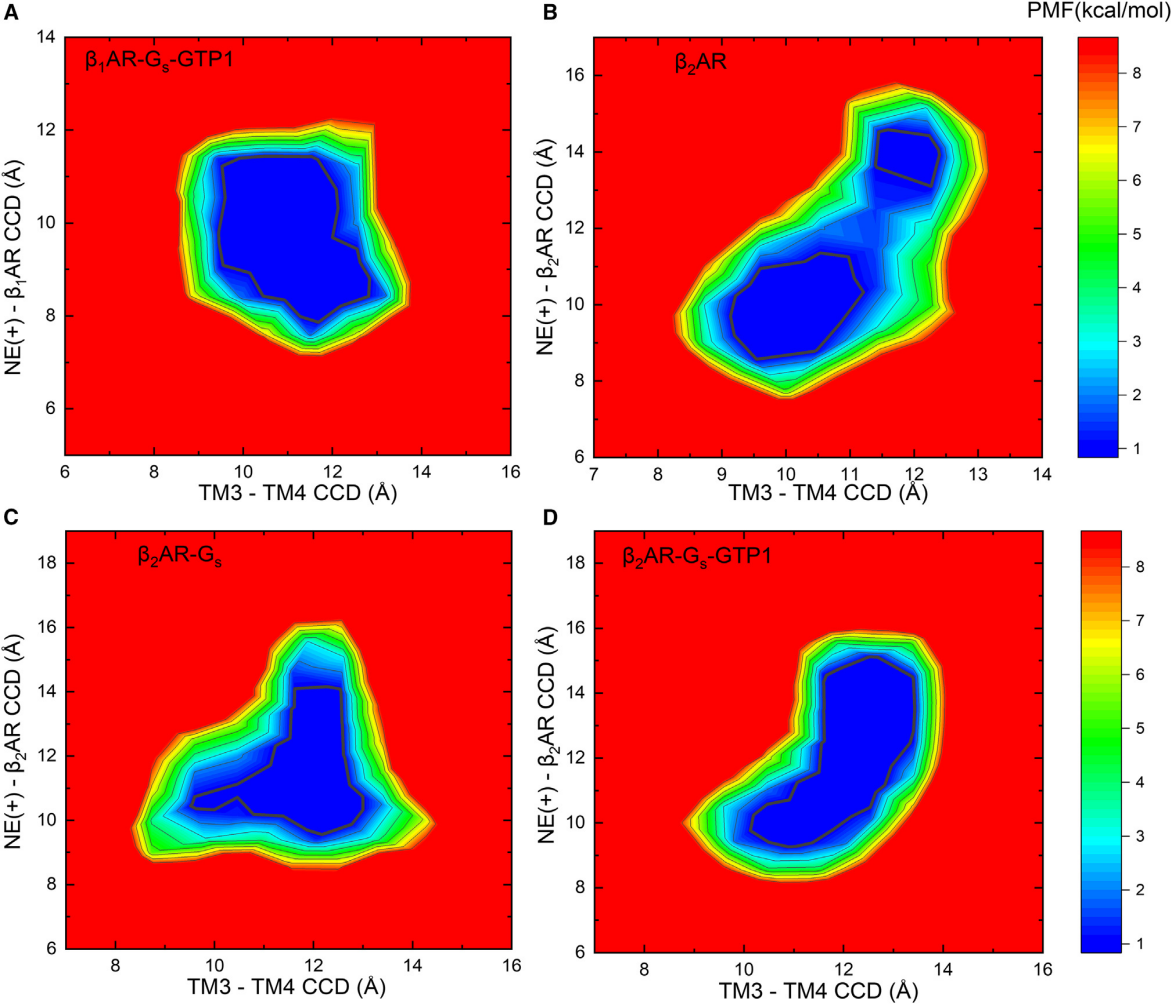
2D PMF or free-energy profiles (in kcal/mol) based on CCD between TM3 and TM4 shown as *x* axis and the CCD between NE(+) and β_1_/β_2_AR shown as *y* axis The distances are measured based on all-atom Anton 2 MD simulations of the following NE(+) bound active state human β_1_AR or β_2_AR systems: (A) β_1_AR-G_s_-GTP1, (B) β_2_AR, (C) β_2_AR-G_s_, and (D) β_2_AR-G_s_-GTP1. 0.5 kcal/mol contour lines are shown as bold black curves. Relative free-energy values from 0 to 8 kcal/mol are indicated by different colors, from blue to red as shown by the color bars on the right. All distances were measured between geometric centers of NE(+), β_1_/β_2_AR, or transmembrane helices. The contour lines are smoothed for better visualization. For the CCDs of TM3 and TM4, the geometric centers of the two helices were used. For the CCD between NE(+) and βAR, the geometric centers of NE(+) and βAR (excluding ICL3 and C-terminal residues) were used.

We computed the 2D potential of mean force (PMF) with respect to the CCD of TM3 and TM4, as well as the CCD between NE(+) and β_1_/β_2_AR, in systems where NE(+) exhibited partial dissociation (Figure 4). For the CCD of TM3 and TM4, the geometric centers of the two helices were used. The CCD between NE (+) and βAR was calculated as described previously in Figure 2. In the β_1_AR-G_s_-GTP1 system, as the distance between TM3 and TM4 decreased, NE(+) demonstrated partial dissociation (Figure 4A). In contrast, in all the β_2_AR systems, NE(+) commenced dissociation when the TM3-TM4 distance increased (Figures 4B–4D). It is worth noting that, in the β_2_AR system (Figure 4B), we observed the presence of two distinct free-energy minima for NE(+), with a barrier of about 1.5 kcal/mol separating them. This barrier disappeared due to the influence of G_s_ and GTP binding (Figures 4C and 4D). Our analysis also suggests that the movement of helices TM3 and TM4 in β_1_AR and β_2_ARs directly alters NE(+) binding. This movement can be related to G_s_ and GTP/GDP binding.

### ECL3 plays a crucial role in determining dissociation pathways of NE from β_1_AR and β_2_AR

**Figure 5:**
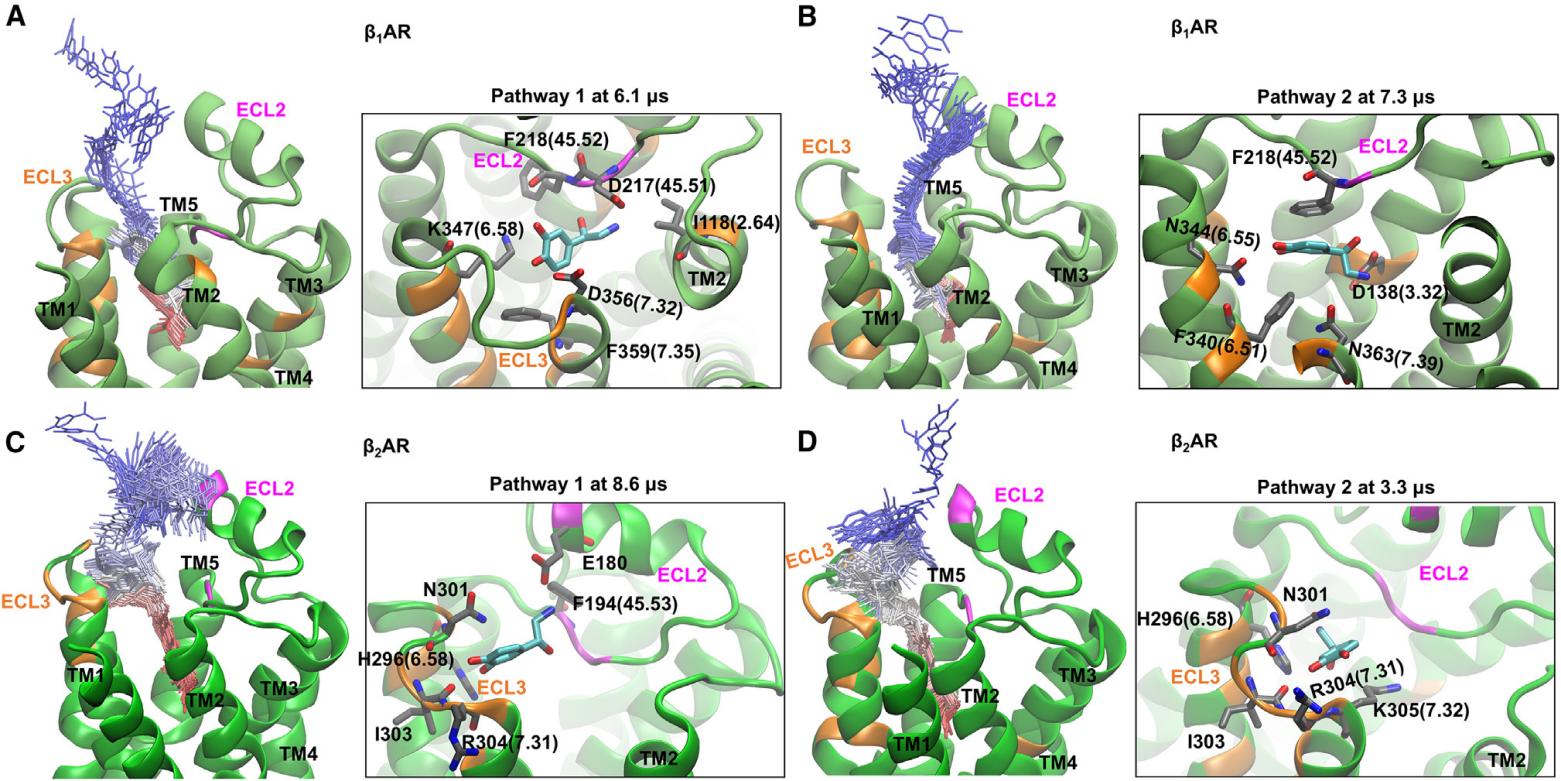
Representative complete NE(+) dissociation pathways captured by the WE method (A) Dissociation pathway 1 of NE(+) in the β_1_AR system with a complete dissociation WE simulation time of 7.5 μs. (B) Dissociation pathway 2 of NE(+) in the β_1_AR system with a complete WE simulation dissociation time of 17.5 μs. (C) Dissociation pathway 1 of NE(+) in the β_2_AR system with a complete WE simulation dissociation time of 12.9 μs. (D) Dissociation pathway 2 of NE(+) in the β_2_AR system with a complete WE simulation dissociation time of 6.7 μs. The pathways were depicted using NE(+) molecules in the wireframe representation transitioning from red through white to blue, representing the progression of the WE simulation time and thus the transition from bound to unbound NE(+) states. The insets, aligned with their corresponding main figures, illustrate the specific β_1_/β_2_AR amino acid residue interactions with NE(+) at certain points in WE simulation time during its dissociation process.

Despite conducting multi-microsecond-long conventional MD simulations using the Anton 2 supercomputer and a microsecond-long GaMD simulation for the NE-β_2_AR system, we were unable to fully sample the dissociation of NE(+) from βAR into the bulk aqueous solution. To investigate the entire dissociation pathway of NE(+), we subsequently performed WE simulations (details can be found in the STAR Methods section). Figure 5 illustrates two representative complete dissociation pathways of NE(+) in β_1_AR and β_2_AR systems. Figures S4–S7 display snapshots captured at various stages of each dissociation pathway. We defined “hot spots” as amino acid residues contributing to NE(+) binding for more than 20% of the dissociation pathway duration. The list of “hotspot” amino acid residues can be found in Tables S7–S10. In Figure 5 the magenta regions indicate residues on ECL2, while the orange regions indicate residues on other loops and TM helices. The shared regions along the NE(+) dissociation pathways in β_1_AR and β_2_AR include TM6, TM7, and ECL2. In β_2_AR, NE(+) typically traverses through ECL3, whereas, in β_1_AR, NE(+) interacts solely with residues on TM6 and TM7 without involving ECL3.

In Figure 5A, captured at 6.1 μs of WE pathway 1 of β_1_AR, NE(+) became ensnared by a gate formed by ionic residues (D45.51, D7.32, K6.58) and aromatic residues (F45.52, F7.35, I2.64) located on TM2, TM6, TM7, and ECL2. Subsequently, NE(+) transitioned past the aromatic residues and interacted solely with the ionic residues, as depicted in Figure S4, captured at 6.4 μs and 6.5 μs of the WE run. A similar phenomenon was found in other WE pathways (Figures 5B–5D) but with different ionic and aromatic residues (Figures S5–S7). These findings underscore the dynamic nature of interacting ionic and aromatic amino acid residues, which may vary over time. The results suggest the presence of allosteric effects, where ECLs of the receptor could influence the conformation or dynamics of residues forming temporary ligand binding pockets, thus impacting ligand affinity. The conserved residue F45.52 on the inner side of ECL2 emerged as a significant feature in both pathways of β_1_AR and one pathway in β_2_AR, highlighting its role in NE(+) dissociation. Similarly, F45.52 was also identified as crucial in the NE(+) association with both β_1_AR and β_2_AR.^36^

In our study, NE(+) was observed to interact with the lid (top helix) of the ECL2 in β_2_AR, highlighted in magenta in Figures 5C and 5D. Specifically, NE(+) interacted with residue E180 in the ECL2 and residues in the ECL3 of β_2_AR (Figure 5C). However, we did not detect a significant interaction between NE(+) and the equivalent residue in the ECL2 of β_1_AR, even though this glutamic acid residue is conserved in both β_1_AR (E205) and β_2_AR (E180). The divergence in NE(+) dissociation pathways between β_1_AR and β_2_AR may be attributed to the region of ECL3, as indicated by our pathways sampled through WE simulations. We observed that NE(+) tends to linger more around ECL3 of β_2_AR (Figures 5C and 5D), whereas it has limited interaction with ECL3 in β_1_AR (Figures 5A and 5B). These suggest that the interaction of NE(+) with ECL3 facilitates its positioning to interact with the top lid of ECL2, specifically residue E180, in β_2_AR. In contrast, the reduced interaction between NE(+) and ECL3 in β_1_AR likely hinders NE(+) from reaching the top of ECL2 in β_1_AR. Figure S2C displays the multiple sequence alignment of β_1_AR and β_2_AR, performed using the Clustal Omega web tool.^55^ The ECL2 region is marked in red, and the ECL3 region in orange. The sequences of ECL3 in β_1_AR and β_2_AR exhibit high variability. The “hotspot” residues around ECL3 for β_2_AR are N301, Y308, H296(6.58), K305, I303, and R304, whereas, in β_1_AR, the "hotspots" are K347(6.58), F359(7.35), D356, and R357. We posit that the higher presence of “hotspots” around ECL3 of β_2_AR may account for NE(+) tending to linger around this loop. In β_1_AR, the “hotspots” are predominantly located toward the inner, membrane-facing side of the helices, whereas, in β_2_AR, they extend to the outer side of ECL2. Our finding suggests that NE(+) allocates a higher percentage of time (out of the total unbinding time) contending within the inner (transmembrane) regions of helices. Once it overcomes obstacles from ionic and aromatic residues on the inner side, it spends less time meandering around the outer side of ECL2 in β_1_AR. Conversely, NE(+) dedicates a greater percentage of time wandering around ECL3 and ECL2 in β_2_AR (Figure 5C and 5D). For β_1_AR, both ECL3 and ECL2 pose minor obstacles during NE dissociation from the orthosteric binding site and its association. However, for β_2_AR, these regions present significant hurdles. The impact of ECL3 and ECL2 is more pronounced in β_2_AR than β_1_AR, given that NE(+) spends more time in these regions in β_2_AR. This observation can explain the decreased ligand association and dissociation rates, *k*_on_ and *k*_off_, of β_1_AR when certain residues in ECL2 and ECL3 are mutated to those in β_2_AR.^36^ The experimental kinetic data can be referenced from Table S1 (Rows 1 and 5 for NE(+) binding β_1_AR) in the study by Xu et al.^36^ As discussed earlier, our GaMD and Anton simulations revealed an additional transient binding site for NE(+) near ECL2 in β_2_AR. In reference to Xu et al.’s experiments,^36^ which suggest that the extracellular vestibule contributes to NE(+) selectivity for β_1_AR over β_2_AR, our simulations further indicate that ECL3 may play a key role in this selectivity.

### G_s_ conformational transitions are more pronounced in β_2_AR systems than β_1_AR systems

**Figure 6:**
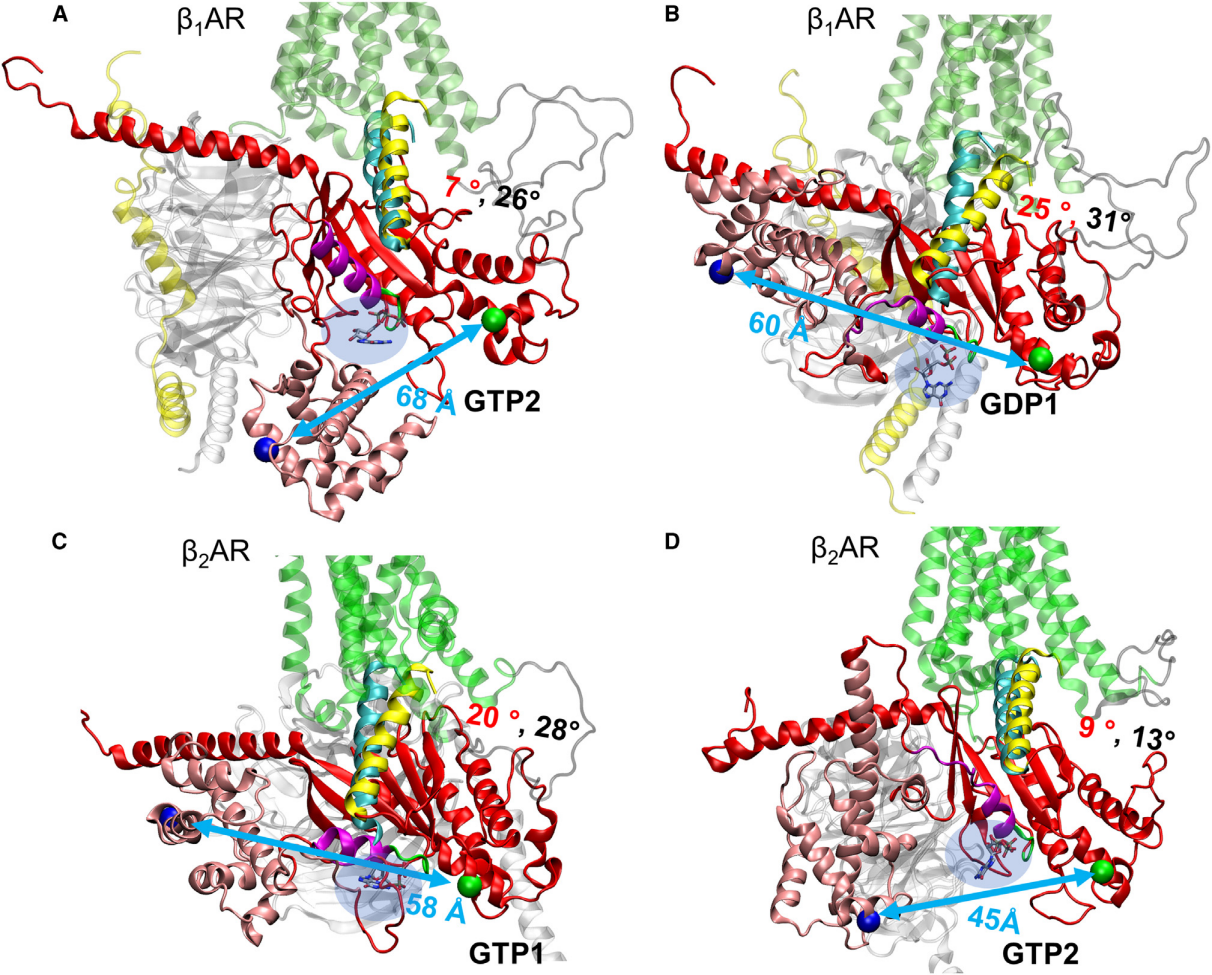
Representative G_s_ protein structures from all-atom Anton 2 MD simulations of the active state human βAR-G_s_ complexes with GTP/GDP bound (A) β_1_AR-G_s_-GTP2. (B) β_1_AR-G_s_-GDP1. (C) β_2_AR-G_s_-GTP1. (D) β_2_AR-G_s_-GTP2. The structures are captured from the 2.5 μs long unbiased MD simulation runs on Anton 2. G_s_α α5 helix is colored in yellow with the initial position in cyan; the angle between the initial and final position of α5 is marked in red, and the maximum angle during the full simulation is marked in black; βAR intracellular loop 3 (ICL3) is colored in gray; α1 helix is colored in purple. Other proteins structural elements are colored as in Figure 1. Residues A161 on the G_s_αAH domain and E299 on the G_s_αRas domain are shown as blue and green balls (C_α_ atoms), respectively, and distances between them are shown by light-blue arrows. GTP/GDP molecules are shown in a light-blue shadow. The common GTP/GDP binding site formed by G_s_α residues K53, S54, G52, E50, and S51 is colored in green.

The conformational dynamics of G_s_α associated with nucleotide exchange were studied separately for both β_1_AR and β_2_AR.^38^^,^^39^^,^^41^^,^^42^ In this study, we examined how guanine nucleotide, GTP or GDP, binding influences the conformational changes of G_s_α when it is bound to β_1_AR or β_2_AR, respectively. We carried out our analysis based on multi-μs-long unbiased MD simulations using Anton 2 and enhanced sampling GaMD simulations. We measured the CCD between residue A161 on AHD and E299 on RD to analyze the conformation change of the G_s_α (Figure 6). If the CCD is greater than or equal to 55 Å, we define G_s_α conformation as fully open; if the distance is in the range of 45–55 Å, we define it as semi-open; if the distance is in the range of 35–45 Å, then it is semi-closed; and, if the distance is less than or equal to 35 Å, then it is a closed conformation, as was defined in our previous study.^54^ The average CCD values over the MD simulation time are 63.6 Å (open) for β_1_AR-G_s_-GTP1, 31.2 Å (closed) for β_1_AR-G_s_-GTP2, 59.7 Å (open) for β_1_AR-G_s_-GDP1, and 62.7 Å (open) for β_1_AR-G_s_-GDP2. Based on the average CCD values, three β_1_AR systems are in open states, and only one is in a closed state.

Regarding the β_2_AR systems, the average CCD values over the MD simulation time are 59.3 Å (open) for β_2_AR-G_s_-GTP1, 42.3 Å (semi-closed) for β_2_AR-G_s_-GTP2, 31.5 Å (closed) for β_2_AR-G_s_-GDP1, and 43.4 Å (semi-closed) for β_2_AR-G_s_-GDP2. Based on the average CCD values, one β_2_AR system is in an open state, and the others show various semi-closed or closed states.

In both β_1_AR and β_2_AR systems, GTP/GDP in position 1 eventually converged to position 2 when bound to G_s_α (Figures S9C and S11C). However, in most cases, GTP/GDP did not remain bound for the entire duration of the MD simulation. We also found that G_s_ conformational transitions from an open to a closed state, as evidenced by CCDs between residue A161 on AHD and E299 on RD, are more obvious in the β_2_AR systems than in the β_1_AR systems, as shown in Anton MD (Figures S8 and S10) and GaMD simulations (Figure S12). Su et al. found that, in the β_1_AR-G_s_ system, a tilting of the α5-helix in G_s_α deformed the GDP/GTP-binding pocket and accelerated GDP release.^39^ Recent time-resolved cryoelectron microscopy studies of the β_2_AR-G_s_ system revealed that the AHD alternates between open and closed states in the nucleotide-free state. GTP binding does not directly induce AHD closure through an allosteric effect; instead, GTP stabilizes the AHD in its closed conformation by locking it against the RD domain, but only after the AHD has randomly adopted the closed state.^56^ Here, we found that, in both βAR systems, GTP/GDP binding can result in randomly occurring G_s_α conformational transitions. It was also noted that, in our previous study of the β_2_AR systems, the conformational change of G_s_α could also occur spontaneously without GTP/GDP binding.^54^ These studies revealed that the conformational transitions of G_s_ from an open to a closed conformation may not be directly related to GTP/GDP binding.

### GTP/GDP binding could influence the G_s_α dissociation

**Figure 7:**
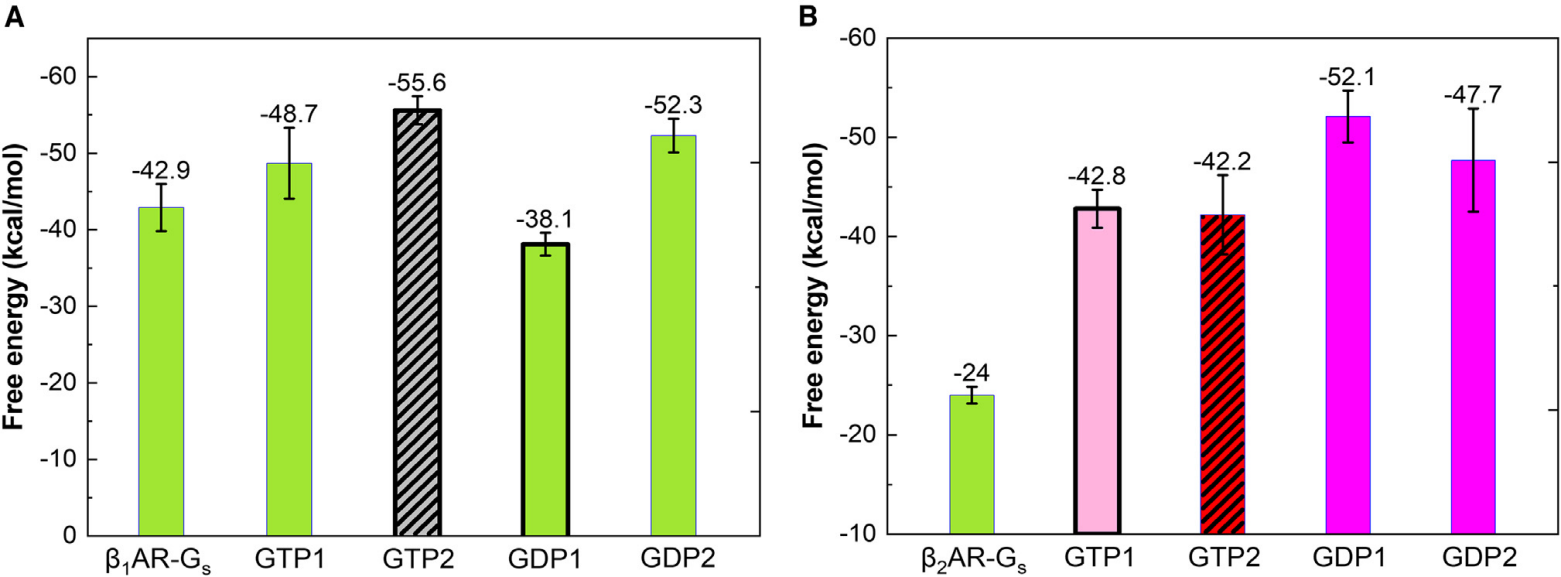
MM-PBSA energies between β_1_AR/β_2_AR and G_s_ α5 with or without GTP/GDP bound (in kcal/mol) using the last 1 μs of Anton 2 MD simulation trajectories (A) for β_1_AR systems and (B) for β_2_AR systems. Standard errors of the mean (SEMs) shown as error bars were computed using block averages. Color codes for the dominant conformation of G_s_ during those simulations: green, open; gray with stripes, semi-open to open; pink, flipped up; red with stripes, open to closed; magenta, semi-closed; bold black frames of the boxes indicate that GTP/GDP was bound during the last 1 μs of those simulations.

Although experimentally GDP was used to promote β_2_AR-G_s_ complex formation, and GTP was used to dissociate the G_s_ from β_2_AR,^57^ the conformation of G_s_ during its dissociation is unclear. We previously found that G_s_α conformational transition is spontaneous, and the open state is more favorable for G_s_α α5 dislocation.^54^ DeVree et al. found that G protein binding and GDP unbinding from G_s_ stabilize the active conformation of the receptor.^58^ Metadynamics simulations also predicted that agonist binding alone does not activate β_2_AR; it needs the binding of GDP-bound G_s_.^59^ To analyze the function of GTP/GDP binding in the dislocation of α5, when G_s_α is in an open state, we calculated the MM-PBSA free energies between G_s_α α5 and βARs (Table S2 and Figures 7A and 7B). We compared the systems with the open G_s_α conformation when GTP or GDP binds. In β_1_AR systems, GDP binding causes a larger α5 tilting angle (Figures S9A and S9B) with a less favorable binding free energy between α5 and β_1_AR (Figure 7A) when comparing GDP-bound β_1_AR-G_s_-GDP1 system to mostly nucleotide unbound β_1_AR-G_s_-GDP2 and β_1_AR-G_s_-GTP1 systems, where such α5 tilting was not observed (Figure S9A). Thus, GDP binding may trigger the G_s_α dissociation from its open state. We cannot compare the GTP versus GDP binding effect in terms of α5 dislocation in β_1_AR systems because we did not detect comparable GTP- and GDP-bound systems with similar G_s_α conformations. Similarly, the conformational flexibilities in the β_2_AR systems make it challenging to find comparable systems for such analysis.

We conducted interdomain correlation analysis for the β_1_AR and β_2_AR systems when GTP/GDP was bound (Tables S11 and S12). We found a strong negative correlation between the α5 tilting angle and the CCD of α1–α5 in both β_1_AR (Pearson correlation coefficient *r* = −0.89) and β_2_AR (*r* = −0.92) systems. Moreover, in the β_1_AR systems with nucleotide bound, a negative correlation was found between the CCD of α1–α5 and that of β_1_AR-α5 (*r* = -0.96). However, when a nucleotide is present in the β_2_AR systems, the strong negative correlation is not preserved (*r* = −0.40), indicating that GTP/GDP altered the initial interdomain correlations, which could influence the G_s_α dissociation.

### GTP/GDP binding to G_s_ disrupts the conformational coupling between NE and βAR and the coupling between βAR and G_s_

To further analyze the impact of G_s_ on NE(+) binding and the effects of GTP/GDP binding on the coupling between NE(+) and βAR and between βAR and G_s_, the RMSDs of NE(+), βAR, and G_s_ conformations from MD simulation trajectory frames were calculated and stored. For NE(+), its conformation with respect to the βAR was used, as well as the conformations of both βAR and G_s_. The machine learning-based clustering technique TTClust^60^ was used to classify frames into clusters based on RMSD. We then compared any clusters between NE(+) and βAR by quantifying the number of common MD frames in both clusters. If a specific pair of clusters (one from NE and the other from βAR) have more than 50% of the common frames, we identify it as a strong coupling pair. Our objective was to identify any potential coupling between NE(+) and βAR conformations and between βAR and G_s_ conformations (resembling key-lock pairs). As shown in Table S13, in the β_1_AR-only system, we found one pair of strong coupling between NE(+) and β_1_AR conformations. No strong coupling pair between NE(+) and β_1_AR poses was found when G_s_ binds without a bound nucleotide. Similar results were observed in β_2_AR systems. This indicates that G_s_ binding changed the original conformational coupling between NE and β_1_AR or β_2_AR.

We also examined the effect of GTP/GDP on the G_s_ binding to β_1_/β_2_AR (Table S13). We observed two strong coupling pairs between β_1_AR and G_s_ conformations in the β_1_AR-G_s_-GTP and four pairs in the β_1_AR-G_s_-GDP systems. In the β_2_AR-containing systems, we observed two strong coupling pairs between β_2_AR and G_s_ conformations in the β_2_AR-G_s_-GTP systems and three pairs in the β_2_AR-G_s_-GDP systems. These results suggest that the binding of GDP to G_s_ promotes more conformational coupling pairs between βAR and G_s_ than GTP binding. On the other hand, we did not observe any NE(+)-β_1_AR coupling pairs in the β_1_AR-G_s_ and β_1_AR-G_s_-GDP complexes, while we found two such pairs in the β_1_AR-G_s_-GTP complexes. Similarly, we found no NE(+)-β_2_AR coupling pairs in β_2_AR-G_s_ and β_1_AR-G_s_-GDP systems and one pair in the β_1_AR-G_s_-GTP systems. These findings suggest that GTP can alter the conformational coupling between NE(+) binding poses and β_1_/β_2_AR conformations to a greater extent than GDP.

**Figure 8:**
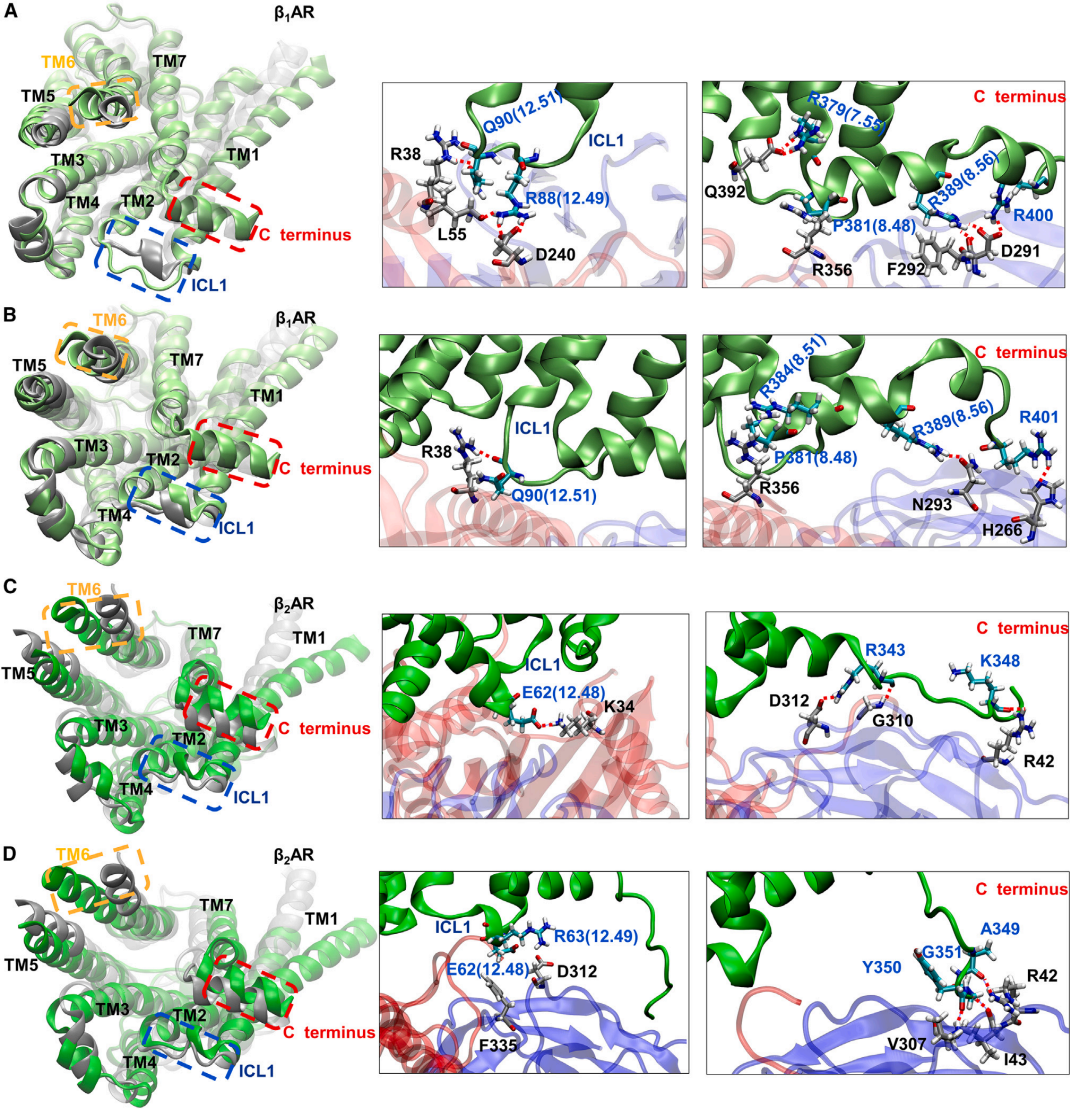
Representative conformations of β_1_AR/β_2_AR when they form mutually selective poses with G_s_ in the following systems (A) β_1_AR-G_s_-GDP1, (B) β_1_AR-G_s_-GDP2, (C) β_2_AR-G_s_-GDP1, and (D) β_2_AR-G_s_-GDP2. Left panels: Representative conformations of the receptor when it forms mutually selective poses with G_s_. Middle panels: the interactions captured between the intracellular loop 1 (ICL1) of βAR and G_s_. Right panels: the interactions captured between the truncated C terminus of βAR and G_s_. βARs are colored in green for their representative conformations, while gray traces represent their initial poses based on PDB structures. ICL3 and the last 7 amino acid residues in the C terminus of βAR were omitted in conformation clustering due to their flexibilities. The terminus of TM6 is shown in an orange dashed box. The truncated C terminus is marked by a red dashed box. The ICL1 in between TM1 and TM2 is in a blue dashed box. The amino acid residues with carbon atoms colored in cyan are from βAR, and those with gray carbon atoms are from G_s_; oxygen atoms are in red; nitrogen atoms are in blue; hydrogen atoms are in white. Amino acid residue names in blue are from βAR, and those in black are from G_s_. Hydrogen bonds are shown as red dashed lines.

Rasmussen et al. found that β_2_AR underwent conformational changes upon interaction with G_s_.^38^ Later, Ma et al. analyzed the β_2_AR-G_s_ and β_2_AR-G_i_ complexes and found that ICL2 may be the key determinant for G protein coupling selectivity.^61^ To assess the distinctions in G_s_ binding between β_1_AR and β_2_AR, we conducted an analysis and comparison of the cytoplasmic sides of β_1_AR and β_2_AR, specifically a region where G_s_ couples, while considering the presence of GDP when conformational coupling pairs were found between βAR and G_s_ (see Figure 8). The C termini of βARs are based on their experimental PDB structures, which are truncated compared to the complete sequences of these proteins. We further truncated the C termini of our MD simulation-derived protein structures for the conformational clustering. Only the portions of C termini with similar α-helical structures were used in β_1_AR- and β_2_AR-containing systems in this clustering, while the entire modeled C termini were shown in the right panels of Figure 8 for residue interaction analysis. The results in the left panels showed obvious differences in three βAR regions: the TM6 highlighted by the orange bounding box, the C terminus highlighted by the red box, and the ICL1 highlighted by the blue box. When coupled to G_s_, β_1_AR in the β_1_AR-G_s_-GDP1 complex exhibited significant movements of ICL1 (Figure 8A), which can be associated with the hydrogen bonds formed between β_1_AR and G_s_ residues: Q90(12.51) and R38 forming one interaction pair, and R88(12.49) interacting with D240 and L55. Less ICL1 movements are found in other βAR systems, as shown in Figure 8B for the β_1_AR-G_s_-GDP2 system and in Figure 8C and 8D for β_2_AR-containing systems β_2_AR-G_s_-GDP1 and β_2_AR-G_s_-GDP2, which can be attributed to fewer or no hydrogen bond pairs (see middle panel of Figures 8B–8D, respectively). β_2_AR tends to exhibit greater movements in the TM6 and C-terminal region than β_1_AR (compare orange and red boxes in the left panels of Figures 8C and 8D with those in Figures 8A and 8B). The C-terminal segment (like an arm) connects with TM7, forming an “elbow joint.” Hydrogen bonding (e.g., R379(7.55)^…^Q392) and hydrophobic interactions (e.g., between P381(8.48) and R356) were found in between the β_1_AR “elbow joint” and G_s_ as shown in the right panels of Figures 8A and 8B. Although no such interactions were found for the “elbow joint” of β_2_AR, there are still hydrogen bonds formed between the very end of the C-terminal segment of β_2_AR (like a hand) and G_s_ (right panels of Figures 8C and 8D). For β_1_AR, both its “elbow joint” and “hand” are “attached” to G_s_, while, for β_2_AR, only the “hand” is “attached” to G_s_, leaving the “arm” free to move. This can be a reason for a larger displacement of the C-terminal segment in β_2_AR compared to β_1_AR during MD simulations. These findings suggest that, even when binding to the same G protein, G_s_, β_1_AR, and β_2_AR exhibit distinct behaviors, as evidenced by the identified regions, including cytoplasmic sides of TM6, ICL1, and C termini. We observed distinct behaviors between β_1_AR and β_2_AR when coupled with the same G protein. This may indicate even greater differences in their coupling with different G proteins such as G_i_, which will be investigated in our future studies.

In addition, we analyzed amino acid residue contacts between ICL3 and G_s_ α5 in both β_1_AR- and β_2_AR-containing systems. The G_s_α α5 helix, which plays a crucial role in forming key contacts with the receptors, is shown in Figure 6 in yellow, and its initial position there is indicated in cyan. Previous computational and experimental studies on the β_2_AR suggest that ICL3 is involved in receptor activation and signaling.^62^^,^^63^^,^^64^ Sadler et al. discovered that ICL3 truncation enhanced signaling for β_2_AR in its G_s_ signaling pathway but not for β_1_AR.^65^ In Table S14, we found that ICL3 directly contributes to interaction with α5 in β_1_AR-containing systems, as was found in β_2_AR systems in our previous study.^54^ However, we did not observe any G_s_α α5 conformational changes besides its tilting when interacting with the ICL3 of β_1_AR, unlike our previous findings in β_2_AR-containing systems.^54^ Our MD simulation results, along with the recent work by Sadler et al.,^65^ suggest that ICL3 may function differently in β_1_AR compared to β_2_AR.

## Discussion

Although β_1_AR and β_2_AR are highly homologous and expressed in the heart, they have distinct roles in regulating cardiac functions.^8^ Research has shown that β_1_AR has a 10-fold higher affinity for NE(+) than β_2_AR.^36^ Metadynamics simulations revealed that NE(+) has different binding (entrance) pathways toward β_1_AR and β_2_AR,^36^ which can explain the different association rates of NE(+) to β_1_/β_2_AR. It was also found that NE(+) can be trapped into a local energy minimum before entering the OBP in β_2_AR.^36^ Our all-atom unbiased multi-microsecond-long MD and enhanced sampling GaMD simulations revealed an NE(+) secondary binding site in β_2_AR but not in β_1_AR during NE(+) partial dissociation, which could explain why NE(+) has a lower experimental affinity for β_2_AR compared to β_1_AR.^36^ In our study, the key amino acid residue that triggers the secondary binding sites for NE(+) in β_2_AR was identified as Y174(4.66), whereas the counterpart residues W199(4.66) and T220(45.54) in β_1_AR are less effective in interacting with NE(+). The residue W199(4.66) possesses an aromatic ring, and T220(45.54) contains a hydroxyl group. This structural configuration suggests that NE(+) may interact with these two amino acid residues in a one-to-two mode. This bifurcated and less constrained interaction mode is potentially less effective, thereby impeding the interaction. Supporting this, recent experimental data demonstrated that conformationally flexible Epi exhibited low selectivity for β_2_AR. In contrast, conformationally constrained Epi showed enhanced affinity for β_2_AR, which may highlight the significance of conformational constraints of the ligand or ligand-receptor interactions as in our simulation system and their impact on selectivity and affinity.^37^

The recent work about the binding pathway of NE to βARs revealed that the extracellular vestibules of the receptors have different shapes and electrostatic properties that influence the path NE takes to the orthosteric binding pocket and contribute to the different association rates and, thus, different affinities.^36^ Unlike that work, we sampled the complete dissociation pathways of NE(+) from β_1_AR and β_2_AR using WE simulations. ECL3, one of the non-conserved βAR regions, was identified as the key region differentiating the NE(+) dissociation pathways. Rather than focusing solely on “binding pathways,” we contend that the key factors influencing the selectivity of NE(+) are the non-conserved regions, particularly those surrounding ECL2 and ECL3 in β_1_AR and β_2_AR. These regions impact binding pathways and play a crucial role in determining dissociation pathways. It is important to note that our WE simulations did not capture the secondary binding sites of NE(+) in β_2_AR, which were identified through conventional multi-microsecond-long unbiased MD and GaMD simulations described in the results section. This discrepancy arises because WE methodology is optimized for pathway sampling, directing the simulation progress toward predefined directions, and thus may not capture the nuanced dynamics observed in conventional and GaMD simulations. Some hybrid methods, such as GaMD combined with WE,^66^ could be used to sample the dynamics and pathways better. Once more, the non-conserved protein regions or individual amino acid residues primarily account for the emergence of the secondary binding site in β_2_AR and consequently influence the selectivity of NE(+). To enhance future high-throughput drug docking and screening strategies, we recommend incorporating the OBP, the non-conserved regions, and the receptor secondary and allosteric ligand binding sites.

ICL3 was absent in the β_2_AR-G_s_ experimental structure,^38^ and most computational receptor structures did not include ICL3.^33^^,^^35^^,^^45^^,^^46^^,^^67^ Our receptor models include all the missing intracellular loops. We found that ICL3 participates in G_s_ interactions in both β_1_AR and β_2_AR, but it produces different effects in each receptor. This indicates the importance of building complete models of receptors with all the missing loops. We also observed distinct TM and intracellular loop movements in the cytoplasmic sides of β_1_AR and β_2_AR when coupled to G_s_. In β_1_AR, ICL1 is involved, while, in β_2_AR, TM6 and the C-terminal regions participate more in G_s_ interactions. Additionally, G_s_ exhibited different behaviors in binding with β_1_AR and β_2_AR, including more frequent conformational transitions of G_s_α between open and closed states in β_2_AR in contrast to a more stable binding of G_s_α with β_1_AR. These findings suggest that β_1_AR and β_2_AR exhibit distinct behaviors even when binding to the same G_s_, as evidenced by the identified regions of βARs, including the cytoplasmic sides of TM6, ICL1, and the C termini.

The conformational dynamics of G_s_α associated with nucleotide exchange were studied extensively, as discussed in the introduction section.^38^^,^^39^^,^^40^^,^^41^^,^^42^^,^^43^^,^^44^^,^^45^^,^^46^ We also found that guanine nucleotide binding affected G_s_α interdomain conformations and triggered the dislocation of α5 of the GDP-bound G_s_ from β_1_AR, whereas the effect was unclear for β_2_AR-G_s_α interaction. However, we are more focused on the impact of GTP/GDP on the overall conformational coupling in the ligand-receptor-G protein systems. Although GTP or GDP does not directly interact with the receptors, GDP caused more conformational coupling between βAR and G_s_ than GTP, while GTP increased conformational coupling between NE(+) and βAR. This further validates the effectiveness of our method incorporating the “key-lock” pairing concept, which can be used as a new parameter to quantify specific receptor–G protein conformational couplings. These findings may help explain the roles of GTP and GDP in regulating βAR and G_s_ interactions and why β_1_AR and β_2_AR may trigger different downstream signaling pathways.^8^^,^^61^

The future work will include the calculation of association (“on”) and dissociation (“off”) rate constants, which can be used to connect atomic-resolution protein models to multiscale functional models of cardiac physiology, as was done in our recent study.^68^ The workflow and methods we used here can be extended to different GPCR subtypes, such as the α_2A_ adrenergic receptor,^69^ as well as biased G_s_/G_i_ signaling.^70^

### Limitations of the study

One limitation of our study is that we only conducted one 2.5 μs long unbiased Anton MD simulation and a 300 ns long GaMD run for each system. In total, we performed 22.4 μs of all-atom MD simulations for eight different systems. We could not repeat or extend those simulations longer due to the limitation of our computational resources. However, our MD simulations were sufficient to observe exciting trends associated with NE, GTP/GDP binding, and G_s_α conformational changes, as discussed earlier. Also, our Anton MD and GaMD simulation results are consistent with each other. Regarding the free-energy computation method, MM-PBSA and other endpoint methods have many limitations.^53^ Thus, estimations of NE(+) affinity may be improved using other methods like alchemical free-energy perturbation or equivalent methods. However, a fairly large simulation system size, complexity, associated computational cost, and possible convergence issues should be taken into account as well.^71^ For the NE-βAR systems, we additionally used WE methodology to sample the full dissociation of NE with 18 μs and 13 μs WE simulations for β_1_AR and β_2_AR systems, respectively. We should note that the WE simulation time cannot reflect the actual physical time because WE accelerates rare event sampling by pruning and duplicating walkers periodically along the chosen progress coordinates.^49^ Thus, based on those simulations alone, we cannot accurately compute the realistic time needed for the NE dissociation. To obtain accurate rate estimates for our system, we would need to run WE for much longer to obtain converged probability fluxes, similar to how “on” and “off” rates in small guest-host systems were obtained from WE simulations.^72^^,^^73^ We will explore a similar approach in the near future, which will be a crucial step for parameter estimation for multiscale functional kinetic models, as discussed earlier. However, obtaining reliable rate constants for large biologically relevant systems in a computationally tractable period currently remains one of the grand challenges in the field. Further characterization of NE dissociation from different binding poses identified in our work would also be of interest, provided sufficient simulation time and available computational resources. Advanced simulation methods for enhanced sampling of biomolecules, such as metadynamics,^50^ umbrella sampling,^74^ adaptive biasing force calculations,^75^ conformational flooding,^76^ and accelerated MD^77^ have been developed over the past few decades. Well-tempered metadynamics, in particular, has proven valuable for studying ligand binding to βARs,^36^^,^^37^ and the activation of those and other GPCRs.^18^^,^^20^^,^^47^ Recent GaMD simulations have been used to study multiple GPCR systems, for instance, capturing intermediate ligand binding states in the chemokine CXCR4 receptor^77^ and the M_3_ muscarinic acetylcholine receptor, as well as full dissociation and binding of the arecoline partial agonist to the M_2_ muscarinic acetylcholine receptor.^78^ However, we are among the first to sample the full dissociation of a small-molecule agonist (NE) from βARs using a statistically unbiased simulation method such as WE. We could not sample the full dissociation of G_s_ protein using WE due to the large size of the G protein and its strong and multiple interactions with the receptor. A new unbiased method based on WE or a related approach could be developed in the near future to sample a large protein dissociation and provide a connection to functional kinetic models for these important subcellular signaling events.

## Resource availability

### Lead contact

Further information and any reasonable requests should be directed to and will be fulfilled by the lead contact, Yanxiao Han (yxhan@ucdavis.edu or hanyanxiao1@gmail.com).

### Materials availability

This study did not generate new unique reagents.

### Data and code availability

• Original simulation data and input files have been deposited at Mendeley Data and are publicly available as of the date of publication at https://doi.org/10.17632/bzmyszrwby.1.
• This paper does not report original code.
• Any additional information required to reanalyze the data reported in this paper is available from the lead contact upon request.

## Acknowledgments

We would like to thank members of the C.E.C. and V.Y.-Y. laboratories for helpful discussions and Prof. Yinglong Miao and his group members for help with GaMD simulations and their analyses. This work was supported by 10.13039/100000968American Heart Association Postdoctoral Fellowship grant 24POST1187017 (to Y.H.); 10.13039/100000002National Institutes of Health Common Fund Grant OT2OD026580 (to C.E.C. and I.V.); 10.13039/100000050National Heart, Lung, and Blood Institute (NHLBI) grants R01HL174001, R01HL128537, R01HL152681, and U01HL126273 (to C.E.C., V.Y.-Y., and I.V.); NHLBI grants R01HL162825, R01HL147263, and VA Merit I01BX005100 and IK6BX005753 (to Y.K.X.); 10.13039/100000050NHLBI grant F31HL174025 (to K.C.R.); 10.13039/100000968American Heart Association Predoctoral Fellowship grant 16PRE27260295 (to K.R.D.); 10.13039/100000968American Heart Association Career Development Award grant 19CDA34770101 (to I.V.); 10.13039/100000001National Science Foundation travel grant 2032486; UC Davis Department of Physiology and Membrane Biology Research Partnership Fund (to C.E.C. and I.V.) as well as UC Davis T32 Predoctoral Training in Pharmacological Sciences fellowship supported in part by NIGMS Institutional Training Grant T32GM099608 (to J.R.D.D.); UC Davis Chemical Biology Program fellowship supported in part by 10.13039/100000057NIGMS Institutional Training Grant T32GM136597 (to K.C.R.); UC Davis T32 Predoctoral Training in Basic and Translational Cardiovascular Medicine fellowship supported in part by 10.13039/100000057NHLBI Institutional Training Grant T32HL086350 (to K.N. and K.C.R.); and the 10.13039/100007707University of California, Davis Department of Chemical Engineering start-up funds (to S.-H.A.). Computer allocations were provided through Advanced Cyberinfrastructure Coordination Ecosystem: Services & Support (ACCESS) and Extreme Science and Engineering Discovery Environment (XSEDE) grant MCB170095 (to I.V., C.E.C., V.Y.-Y., and K.R.D.); 10.13039/100010548National Center for Supercomputing Applications (NCSA); Texas Advanced Computing Center (TACC) Leadership Resource and Pathways Allocations MCB20010 (to I.V., C.E.C., V.Y.-Y., S.-H.A., and K.R.D.); Oracle cloud credits award and Oracle for Research fellowship (to I.V. and C.E.C.); and Pittsburgh Supercomputing Center (PSC) Anton 2 allocations PSCA17085P, MCB160089P, PSCA18077P, PSCA17085P, and PSCA16108P (to I.V., C.E.C., V.Y.-Y., and K.R.D.). Anton 2 computer time was provided by the Pittsburgh Supercomputing Center (PSC) through grant R01GM116961 from the 10.13039/100000002National Institutes of Health. The Anton 2 machine at PSC was generously made available by D.E. Shaw Research.

## Author contributions

I.V., Y.H., S.-H.A., V.Y.-Y., C.E.C., and Y.K.X. designed the research study; Y.H., J.R.D.D., K.R.D, K.C.R., K.N., and S.B. conducted simulations, acquired and analyzed data; all authors wrote the manuscript.

## Declaration of interests

The authors declare no competing interests.

## Declaration of generative AI and AI-assisted technologies

While preparing this manuscript, the authors used ChatGPT to proofread the text for grammar and punctuation. After using this tool, the authors reviewed and edited the content as needed and took full responsibility for the publication’s content.

## STAR★Methods

### Key resources table

**Table:** 

REAGENT or RESOURCE	SOURCE	IDENTIFIER
**Deposited data**		

Title: "Data for Molecular simulations reveal intricate coupling between agonist-bound β-Adrenergic Receptors and G Protein". This dataset includes key input, output, and parameter files, as well as short trajectories for the systems we simulated in this study.	Mendeley Data	https://data.mendeley.com/datasets/bzmyszrwby/1

**Software and algorithms**		

UCSF Chimera		https://www.cgl.ucsf.edu/chimera/
Rosetta ligand docking	Rosetta software suite	https://rosettacommons.org/software/download/
CHARMM-GUI		https://www.charmm-gui.org/
NAMD		https://www.ks.uiuc.edu/Research/namd/
Anton 2	Pittsburgh Supercomputing Center/DE Shaw Research	https://www.psc.edu/resources/anton-2/
WESTPA 2.0		https://westpa.readthedocs.io/en/latest/
Amber		https://ambermd.org/
MM-PBSA	Amber Tools	https://ambermd.org/AmberTools.php
GaMD		https://www.med.unc.edu/pharm/miaolab/resources/gamd/
VMD		https://www.ks.uiuc.edu/Research/vmd/
LPATH		https://lpath.readthedocs.io/en/latest/
Clustal Omega web tool		https://www.ebi.ac.uk/jdispatcher/msa/clustalo
TTClust		https://github.com/tubiana/TTClust

### Method details

#### Protein structures

The 3D coordinates of adrenaline-bound β_2_AR were obtained from the published X-ray crystallographic structure (PDB: 4LDO) to serve as a template for the active receptor.^79^ The G_s_ heterotrimer template was obtained from the 3D coordinates of the crystal structure of the β_2_AR-G_s_ complex bound to agonist P0G (PDB: 3SN6).^38^ 3D coordinates were oriented via the Orientations of Proteins in Membranes (OPM) database.^80^ The adrenaline-bound receptor from PDB: 4LDO was aligned to the protein complex structure from PDB: 3SN6 using UCSF Chimera^81^ Matchmaker to replace the P0G-bound receptor of 3SN6; then all ligands and non-native proteins were removed. The resulting template, which combined the receptor of 4LDO with the G_s_ heterotrimer of 3SN6, was then assessed for clashing van der Waals radii before proceeding. Details can be found in our previous work.^54^

Xu et al. published crystal structures of the human β_1_AR in complex with epinephrine (PDB: 7BU6) and a nanobody.^36^ This structure was selected as a template for the active state model of β_1_AR. The previously modeled G_s_ heterotrimer derived from PDB structure 3SN6 was used to form a human β_1_AR-G_s_ complex template. All model coordinates were obtained as biological assemblies oriented by the Orientations of Proteins in Membranes (OPM) database^80^ but were subsequently aligned to the previously developed β_2_AR-G_s_ model and cleaned of ligands and all non-native peptides using UCSF Chimera to ensure consistent orientation.^80^^,^^81^ The human β_1_AR-G_s_ complex was assessed for steric clashes using van der Waals radii before proceeding to loop modeling. Details can be found in our previous work.^82^

#### Molecular docking

ROSETTA-Ligand^83^ was used for all NE(+) and GTP/GDP docking simulations. Ligand rotamers and parameters were generated by OpenEye Omega^84^ and ROSETTA scripts. The crystal structure of the closed-state G_s_α-GTPγS (PDB: 1AZT),^85^ which is not bound to a receptor, shows that GTP is enclosed between Ras-like GTPase domain (RD) and the α-helical domain (AHD) domains. However, our starting simulation structure is an open G_s_α. To check the effect of GTP/GDP binding on the conformational change of G_s_α, the GTP or GDP molecule was placed at two positions near the RD or AHA domain of G_s_α. A box size of 5 Å was used for ligand transformations along with 7 Å ligand distance cutoff for side chain and backbone reorientations (with <0.3 Å protein backbone C_α_ restraint). 50,000 structures were generated in each run, with the top 10% selected by total score, out of which the lowest interfacial score structures were chosen.

#### Molecular dynamics simulations

MD simulation systems of ∼222,000 or ∼302,000 atoms were generated using CHARMM-GUI^86^^,^^87^^,^^88^ and consisted of βAR protein or βAR-G_s_ protein complex in lipid bilayers soaked by a 0.15 M NaCl aqueous solution. The outer bilayer leaflet contained pure 1-Palmitoyl-2-oleoylphosphatidylcholine (POPC), whereas the inner leaflet had ∼70% POPC and ∼30% 1-Palmitoyl-2-oleoylphosphatidylserine (POPS) as in a previous MD simulation study.^40^ The same ionizable protein residue protonation states, post-translational modifications (lipidations and disulfide bonds based on UniProt data), and C- and N- protein termini were used as in that study.^40^ All-atom biomolecular CHARMM36m protein,^89^ C36 lipid,^90^ and general CHARMM (CGENFF) small-molecule ligand^91^ force fields and TIP3P water^92^ were used. CGENFF program^93^^,^^94^ was used to generate cationic norepinephrine, NE(+), force field parameters by analogy, which were validated and had to be optimized for one dihedral angle using an established quantum-mechanics (QM) based protocol.^91^ Optimized NE(+) parameters are available from our previous study.^54^ For GDP and GTP, CGENFF small-molecule ligand^91^ force fields were used.

MD simulations were run in the *NPT* ensemble at 310 K and 1 atm pressure using a tetragonal periodic boundary condition. The systems were equilibrated for 90 ns, gradually reducing restraints on the protein atoms in the first 40 ns, using the NAMD program.^95^ The equilibrated runs were then followed by multi-microsecond long production runs on the Anton 2 supercomputer^96^ or using enhanced sampling Gaussian accelerated MD (GaMD)^48^ runs, respectively.

#### Gaussian accelerated MD simulations

GaMD is an enhanced sampling method for MD simulations that can efficiently sample thermodynamic properties, such as the free-energy landscape of the system, by adding a boost potential to the energy function of the system.^48^ The GaMD module implemented in the NAMD^97^ was applied to perform GaMD simulations, which included a 10-ns short conventional MD (cMD) simulation (after the previous 90 ns MD equilibration), used to collect potential statistics for calculating the GaMD acceleration parameters, 50-ns GaMD equilibration after adding the boost potential, and finally, a 300-ns GaMD production run. For the β_2_AR system, an extended GaMD run up to 2.5 μs was performed. All GaMD simulations were run at the “dual boost” level, boosting both total and dihedral potential energies by setting the reference energy to the lower bound. The upper limit of the boost potential standard deviation (SD), σ_0_ was set to 6.0 kcal/mol for both the dihedral and the total potential energy terms.

#### Weighted ensemble MD simulations

The weighted ensemble method (WE) is another enhanced sampling method that runs an ensemble of parallel trajectories with probabilities or “weights” and uses a statistical resampling strategy of replicating and pruning trajectories to focus computational effort on difficult-to-sample regions.^98^ More details can be found in the original WE work,^99^ a review article,^49^ and the weighted ensemble simulation toolkit with parallelization and analysis (WESTPA) publications.^100^^,^^101^^,^^102^ There are two types of WE simulations: equilibrium and steady-state WE. In our simulations, we employed equilibrium WE to investigate the dissociation pathways of NE(+). Our selection of progress coordinates included the center-to-center distance (CCD) between NE(+) and βAR, as well as the root-mean-square deviation (RMSD) of NE(+). The bound state was defined as having a center-to-center distance (CCD) smaller than 12 Å, whereas the unbound state was defined by a CCD greater than 40 Å. To automate the placement of bins along the chosen progress coordinate during WE simulations, we implemented the minimal adaptive binning (MAB) scheme in WESTPA 2.0 software.^102^ The resampling interval τ was set to be 50 ps. Our WE simulation ran many unbiased MD trajectory segments in parallel using the Amber MD engine,^103^^,^^104^ with each segment halted and examined after 50 ps. After each interval, trajectories are either replicated or pruned based on a predefined criterion of trajectory count (8 trajectories per bin). This triggers automated adjustment of trajectory weights to facilitate the completion of the resampling process. Since we used the Amber program as the MD engine during the WE simulation, we converted the CHARMM forcefields used in the Anton MD and GaMD simulations to the Amber program compatible forcefield format. We identified 2,299 NE(+) dissociation pathways within approximately 18 μs of WE simulation for the β_1_AR system and 270 pathways within approximately 13 μs of WE simulation for the β_2_AR system. Using the LPATH (linguistic pathway analysis of trajectories) Python tool,^105^ pathways were clustered into two groups for each βAR case. Subsequently, two representative pathways with the highest weights were chosen for further analysis within each βAR system.

#### MD simulation analyses

MD simulation analyses were performed using the VMD program^106^ and lab-generated codes. The potential of mean force (PMF) profiles were calculated based on the probability of the variables using the Boltzmann inversion.^107^ A bin size of 1.0 Å was used for the interatomic distances. The cutoff was set to 10 configurations in one bin for 2D PMF calculations. For instance, 2D PMFs in Figure 4 were estimated from the probability densities of specific system conformations based on MD simulation trajectories along the selected reaction coordinates.

#### MM-PBSA binding energy calculations

Free energy calculations for βAR-NE(+) binding and βAR-G_s_α5 binding were performed using the Molecular Mechanics Poisson-Boltzmann Surface Area (MM-PBSA) approach with all-atom simulation trajectories by MMPBSA.py program in Amber Tools.^108^ The Chamber module of the ParmEd program was used to convert CHARMM-style forcefields to Amber-style forcefields.^109^ The aqueous solution with ionic strength of 150 mM and lipid membrane were treated implicitly using dielectric constants (water ε_w_ = 80, lipid membrane ε_l_ = 2, and protein ε_p_ = 4). The solvent probe radius is set to 1.4 Å, and the atomic radii were set according to the converted force field parameters. To obtain the enthalpy (Δ*H*) contributions of solvation and gas-phase free energies, the particle-particle particle-mesh (P3M) procedure was used.^110^ These calculations were performed with an implicit membrane, where the electrostatic energy includes both reaction field and Coulombic electrostatic energies. Entropy was calculated separately by the interaction entropy method.^111^ This method was shown to increase the entropy calculation efficiency and possibly improve the accuracy of MM-PBSA in estimating protein-protein interactions.^112^ To use the interaction entropy method, gas-phase interaction energies, including Coulombic electrostatic and van der Waals components, were computed. To get the gas-phase Coulombic energy separated from the reaction field energy contribution, each system energy was recalculated using the dielectric boundary surface charges method in the implicit solution. In this study, we focused on trends in relative binding free energies for the same or similar (βAR and βAR-G_s_) protein systems, which may justify the usage of a standard MM-PBSA approach^108^ along with interaction entropy calculations.^111^

#### Binding poses clustering

The clustering for the NE(+) binding poses, βAR conformations, and G_s_ conformations were performed by the TTClust program.^60^ For clustering of NE(+) poses, the trajectories of NE(+) were first aligned to the βAR protein (without intracellular loop 3) in the first frame. The RMSDs of NE(+) between all pairs of frames were calculated and stored in a matrix. This matrix was then used to calculate a linkage matrix using the hierarchical cluster linkage function of the SciPy package.^113^ Ward’s method within the SciPy module was used to minimize the variance within clusters and allow more demarcated clusters to be obtained.^60^ K-means clustering with the Elbow algorithm was used to find the optimal number of clusters.^60^ To cluster the β_1_/β_2_AR conformations, the trajectories of β_1_/β_2_AR without intracellular loop 3 were aligned to their respective reference PDB structures. To cluster the G_s_ conformations, the trajectories of G_s_ were aligned to the PDB structures of β_1_/β_2_AR. The same protocols were then followed for the NE(+) clustering.

### Quantification and statistical analysis

There are no quantification or statistical analyses to include in this study.
