# Supplementary material for: Molecular simulations reveal intricate coupling between agonist-bound β-adrenergic receptors and G protein
Source: iScience. 2025 Jan 2;28(2):111741. doi: 10.1016/j.isci.2024.111741 (PMC11787599; doi:10.1016/j.isci.2024.111741)
Supplement: Document S1. Figures S1–S15, Tables S1–S14 [file mmc1.pdf]

iScience, Volume *28*

Supplemental information

Molecular simulations reveal intricate coupling between agonist-bound β-adrenergic receptors and G protein

Yanxiao Han, John R.D. Dawson, Kevin R. DeMarco, Kyle C. Rouen, Khoa Ngo, Slava Bekker, Vladimir Yarov-Yarovoy, Colleen E. Clancy, Yang K. Xiang, Surl-Hee Ahn, and Igor Vorobyov

***Table S1**. MM-PBSA binding free energies (ΔG) as well as their enthalpic (ΔH) and entropic contributions (-TΔS) (in kcal/mol) between NE(+) and βAR, based on the last 1 μs of Anton 2 MD simulation trajectories. Standard errors of mean (SEM) were computed using block averages.*

System	Time	Δ*H*	-*T*Δ*S*	Δ*G* ± SEM
β_1AR	1.5 – 2.5 μs	-25.40	5.22	-20.18 ± 0.68
β_1AR-G_s	1.5 – 2.5 μs	-27.78	8.02	-19.76 ± 1.38
β_1AR-G_s-GTP1	1.5 – 2.5 μs	-27.43	6.45	-20.98 ± 0.61
β_1AR-G_s-**GTP2**[1]	1.5 – 2.5 μs	-27.49	6.68	-20.81 ± 0.68
β_1AR-G_s-**GDP1**[2]	1.5 – 2.5 μs	-27.47	8.54	-18.93 ± 0.54
β_2AR	1.5 – 2.5 μs	-21.61	6.88	-14.73 ± 0.92
β_1AR-G_s-GDP2	1.5 – 2.5 μs	-25.45	6.76	-18.69 ± 0.77
β_2AR-G_s-**GTP1**[3]	1.5 – 2.5 μs	-25.05	8.36	-16.69 ± 1.51
β_2AR-G_s-GTP2[4]	1.5 – 2.5 μs	-25.48	6.99	-18.49 ± 0.74
β_2AR-G_s-GDP1[5]	1.5 – 2.5 μs	-24.19	9.29	-14.90 ± 0.64
β_2AR-G_s-GDP2[5]	1.5 – 2.5 μs	-28.02	7.65	-20.37 ± 0.61

[1] Semi-open to open G_s conformation with GTP bound (shown in bold).
[2] Open G_s conformation with GDP bound (shown in bold).
[3] G_s AHD flips up with GTP bound (shown in bold).
[4] Open to closed G_s conformation.
[5] Semi-closed G_s conformation.

Table S2*. MM-PBSA binding free energies (ΔG) as well as their enthalpic (ΔH) and entropic contributions (-TΔS) (in kcal/mol) between β_1AR/β_2AR and G_s α5 with or without GTP/GDP bound, using last 1 μs of Anton 2 MD simulation trajectories. Standard errors of mean (SEM) were computed using block averages.*

System	Time	**Δ*H***	**-*T*Δ*S***	**ΔG ± SEM**
β_1AR-G_s	1.5 – 2.5 μs	-70.0	27.1	-42.9±3.08
β_1AR-G_s– GTP1	1.5 – 2.5 μs	-80.2	31.5	-48.7±4.63
β_1AR-G_s – **GTP2**[1]	1.5 – 2.5 μs	-84.6	29.0	-55.6±1.84
β_1AR-G_s – **GDP1**[2]	1.5 – 2.5 μs	-74.7	36.6	-38.1±1.49
β_1AR-G_s – GDP2	1.5 – 2.5 μs	-82.4	30.1	-52.3±2.19
β_2AR-G_s	2.5 – 5 .0 μs	-65.4	41.4	-24.0±0.85
β_2AR-G_s – **GTP1**[3]	1.5 – 2.5 μs	-75.0	32.2	-42.8±1.92
β_2AR-G_s – GTP2[4]	1.5 – 2.5 μs	-82.9	40.7	-42.2±3.99
β_2AR-G_s – GDP1[5]	1.5 – 2.5 μs	-82.7	30.6	-52.1±2.62
β_2AR-G_s – GDP2[5]	1.5 – 2.5 μs	-79.7	32.0	-47.7±5.20

[1] Semi-open to open G_s conformation with GTP bound (shown in bold).
[2] Open G_s conformation with GDP bound (shown in bold).
[3] G_s AHD flips up with GTP bound (shown in bold).
[4] Open to closed G_s conformation.
[5] Semi-closed G_s conformation.

Table S3. *The amino acid residues of β_1AR, which contribute to NE(+) binding for a percentage of the simulation time of the β_1AR-G_s-GTP1 system Anton 2 MD run, solely during periods when the distance between the ligand and the receptor centers of geometry exceeds 10 Å. Residues in bold are highlighted in main-text Figure 2A.*

Residues	% of time
N363(7.39)	100
D138(3.32)	100
S228(5.42)	98.4
V139(3.33)	97.2
F340(6.51)	96.1
N344(6.55)	95.6
**F218(45.52)**	93.5
**T220(45.54)**	84.3
**W199(4.66)**	54.6
A225(5.39)	51.2

Table S4. *The amino acid residues of β_2AR, which contribute to NE(+) binding for a percentage of the simulation time of the β_2AR system Anton 2 MD run, solely during periods when the distance between the ligand and the receptor centers of geometry exceeds 12 Å. Residues in bold are highlighted in the main-text Figure 2B.*

Residues	% of time
D113(3.32)	100
N312(7.39)	95.2
**F193(45.52)**	93.3
T110(3.29)	87.2
V114(3.33)	78.9
**Y174(4.66)**	70.3
Y316(7.43)	57.5
T199(5.38)	51.8
R175(4.67)	48.9
**F194(45.53)**	40.3

***Table S5**. The amino acid residues of β_2AR, which contribute to NE(+) binding for a percentage of the simulation time of the β_2AR system GaMD run 1, solely during periods when the distance between the ligand and the receptor centers of geometry exceeds 12 Å. Residues in bold are highlighted in the main-text Figure 2C.*

Residues	% of time
T110(3.29)	86.6%
**F193(45.52)**	80.3%
**Y174(4.66)**	67.8%
**T195(45.54)**	54.7%
D113(3.32)	50.8%
**F194(45.53)**	50.8%
D192(45.51)	47.0%
Y185	46.4%
F289(6.51)	45.6%
C191(45.50)	40.6%
N312(7.39)	40.3%

***Table S6**. The Pearson Correlation coefficients (r) between root-mean-square deviation (RMSD) of each transmembrane (TM) helix and RMSD of NE(+) in Anton 2 MD runs of different βAR containing systems.*

Helix \ system	β_1AR-G_s-GTP1	β_2AR	β_2AR-G_s	β_2AR-G_s-GTP1
TM1	0.15	-0.16	0.52	0.06
TM2	0.47	0.46	0.22	0.06
TM3	0.58	0.57	0.73	0.19
TM4	0.51	0.87	0.39	0.14
TM5	-0.21	0.24	0.07	-0.37
TM6	0.42	0.17	0.59	-0.07
TM7	0.44	0.22	0.48	-0.02

Table S7. *The amino acid residues of β_1AR that contribute to NE(+) binding for a percentage of the complete ligand dissociation MD simulation time in pathway 1 of β_1AR weighted ensemble (WE) run.*

Residues	% of time
F218(45.52)	76.3%
D356(7.32)	58.9%
F359(7.35)	56.5%
K347(6.58)	52.2%
D217(45.51)	52.2%
D138(3.32)	32.4%
F340(6.51)	30.0%
W134(3.28)	29.0%
N344(6.55)	28.0%
I118(2.64)	26.1%
N363(7.39)	20.3%

Table S8. *The amino acid residues of β_1AR that contribute to NE(+) binding for a percentage of the complete ligand dissociation MD simulation time in pathway 2 of β_1AR weighted ensemble (WE) run.*

Residues	% of time
F218(45.52)	76.1%
D138(3.32)	75.6%
F340(6.51)	71.4%
F359(7.35)	69.2%
N363(7.39)	69.1%
N344(6.55)	42.2%
D356(7.32)	40.1%
V139(3.33)	35.7%
S228(5.42)	32.5%
V360(7.36)	22.1%
R357(7.33)	22.0%

Table S9*. The amino acid residues of β_2AR that contribute to NE(+) binding for a percentage of the complete ligand dissociation MD simulation time in pathway 1 of β_2AR weighted ensemble (WE) run.*

Residues	% of time
N301(ECL3)	58.2%
H296(6.58)	56.2%
K305(7.32)	54.4%
Y308(7.35)	51.0%
I303(ECL3)	51.0%
F195(45.54)	47.9%
F196(5.35)	39.5%
R304(7.31)	28.9%
E180(ECL2)	28.4%
N293(6.55)	19.0%
F289(6.51)	16.7%

Table S10. *The residues of β_2AR that contribute to NE(+) binding for a percentage of the complete ligand dissociation MD simulation time in pathway 2 of β_2AR weighted ensemble (WE) run.*

Residues	% of time
N301(ECL3)	50.2%
Y308(7.35)	49.8%
H296(6.58)	44.2%
F193(45.52)	39.1%
N293(6.55)	38.7%
K305(7.32)	36.9%
F289(6.51)	34.1%
E180(ECL2)	30.1%
F194(45.53)	27.6%
D113(3.32)	26.3%
I303(7.30)	23.5%
R304(7.31)	21.7%
N312(7.39)	21.2%

Table S11. *Pearson correlation coefficients (r) calculated for any two geometric criteria characterized for the GTP/GDP binding cases based on Anton 2 MD runs of* β_1*AR containing systems:* ***A*** *–* $G_s\alpha$ *A161 to E299 distance,* ***B*** *– angle between two vectors of* $G_s\alpha$*AH (AHD) and* $G_s\alpha$*Ras (RD) domains,* ***C*** *–* $G_s\alpha$*AH and* $G_s\alpha$*Ras interdomain distance,* ***D*** *–* β_1*AR NpxxY to* $G_s\alpha$ α*5 distance,* ***E*** *–* β_1*AR to* $G_s\alpha$ *α5 distance,* ***F*** *–* $G_s\alpha$ *α1 to α5 distance,* ***G*** *– α5 tilting angle. A161–E299 distances indicate the* G_s *protein conformational change (opening or closing), while AHD–RD distances show relative movement between those two domains. The angle between the two vectors of AHD and RD domains indicates their relative orientation. α1–α5 distances indicate relative movement between α1 and α5 helices in* $G_s\alpha$*. βAR–α5 distances indicate partial dissociation of* $G_s\alpha$ *α5 helix from the receptor. βAR NPxxY motif–α5 helix distances also indicate* $G_s\alpha$ *α5 partial dissociation. α5 tilting angle is the angle between the initial and final position of α5 during an MD simulation. The Pearson correlation coefficients (r values) were calculated among the data points in Figures S8 and S9A collected from the time periods when GTP/GDP was bound to* G_s *in each Anton 2 run.*

A and F	B and F	C and F	D and F	**E and F**	
-0.01	-0.10	0.75	-0.99	-0.96	
A and G	B and G	C and G	D and G	**E and G**	**F and G**
0.46	0.54	-0.97	0.82	0.72	-0.89

Table S12. *Pearson correlation coefficients (r) calculated for any two geometric criteria characterized for the GTP/GDP binding cases based on Anton 2 MD runs of* β_2*AR containing systems:* ***A*** *–* $G_s\alpha$ *A161 to E299 distance,* ***B*** *– angle between two vectors of* $G_s\alpha$*AH and* $G_s\alpha$*Ras domains,* ***C*** *–* $G_s\alpha$*AH and* $G_s\alpha$*Ras interdomain distance,* ***D*** *–* β_2*AR NpxxY to* $G_s\alpha$ α*5 distance,* ***E*** *–* β_2*AR to* $G_s\alpha$ *α5 distance,* ***F*** *–* $G_s\alpha$ *α1 to α5 distance,* ***G*** *– α5 tilting angle. The geometric criteria were defined identically to those listed in Table S11. The Pearson correlation coefficients (r values) were calculated among the data points in Figures S10 and S11A collected from the time periods when GTP/GDP was bound to* G_s *in each Anton 2 run.*

A and F	B and F	C and F	D and F	**E and F**	
-0.69	-0.80	0.67	-0.46	-0.40	
A and G	B and G	C and G	D and G	**E and G**	**F and G**
0.90	0.96	-0.71	0.71	0.73	-0.92

Table S13. *The number of mutual selection pairs in each system based on Anton 2 MD run. Please see the main-text Methods section for explanation on how these pairs were identified.*

System	Coupling	Mutual selection pairs
β_1AR	NE and β_1AR	**1**
β_1AR-G_s	NE and β_1AR	**0**
	β_1AR - G_s	**1**
β_1AR-G_s-GTP	NE and β_1AR	**2**
	β_1AR - G_s	**2**
β_1AR-G_s-GDP	NE and β_1AR	**0**
	β_1AR - G_s	**4**
β_2AR	NE and β_2AR	**2**
β_2AR-G_s	NE and β_2AR	**0**
	β_2AR - G_s	**3**
β_2AR-G_s-GTP	NE and β_2AR	**1**
	β_2AR - G_s	**2**
β_2AR-G_s-GDP	NE and β_2AR	**0**
	β_2AR - G_s	**3**

***Table S14**. Amino acid residue (AA) contact information from Anton 2 MD runs. (Close contacts are defined as any atom of AAs that is within 3 Å of each other. The stable contacts are defined as AA interacting more than 50% of the MD simulation time. The average percentage interaction time was calculated by averaging the interaction times of the stable AA contacts in the third column).*

Contacts		Number of stable contacts (β_1AR, β_2AR)	Average percentage interaction time (β_1AR, β_2AR)
AA in β_1AR/β_2AR (no ICL3) interact with $G_s\alpha$ α5	GTP1	15, 15	87.3, 89.3
	GTP2	15, 17	93.1, 86.7
	GDP1	14, 16	90.4, 90.5
	GDP2	15, 18	91.0, 84.0
AA in $G_s\alpha$ α5 interact with β_1AR/β_2AR (no ICL3)	GTP1	12, 13	96.1, 93.7
	GTP2	13, 15	93.2, 94.1
	GDP1	11, 13	96.8, 94.6
	GDP2	13, 13	95.5, 94.0
AA in β_1AR/β_2AR ICL3 interact with $G_s\alpha$ α5	GTP1	4, 3	84.9, 90.6
	GTP2	3, 3	85.3, 74.6
	GDP1	3, 3	86.6, 93.3
	GDP2	4, 1	87.5, 89.1
AA in $G_s\alpha$ α5 interact with β_1AR/β_2AR ICL3	GTP1	3, 3	96.9, 93.6
	GTP2	3, 3	93.4, 85.4
	GDP1	3, 3	86.9, 94.6
	GDP2	4, 1	89.1, 86.5

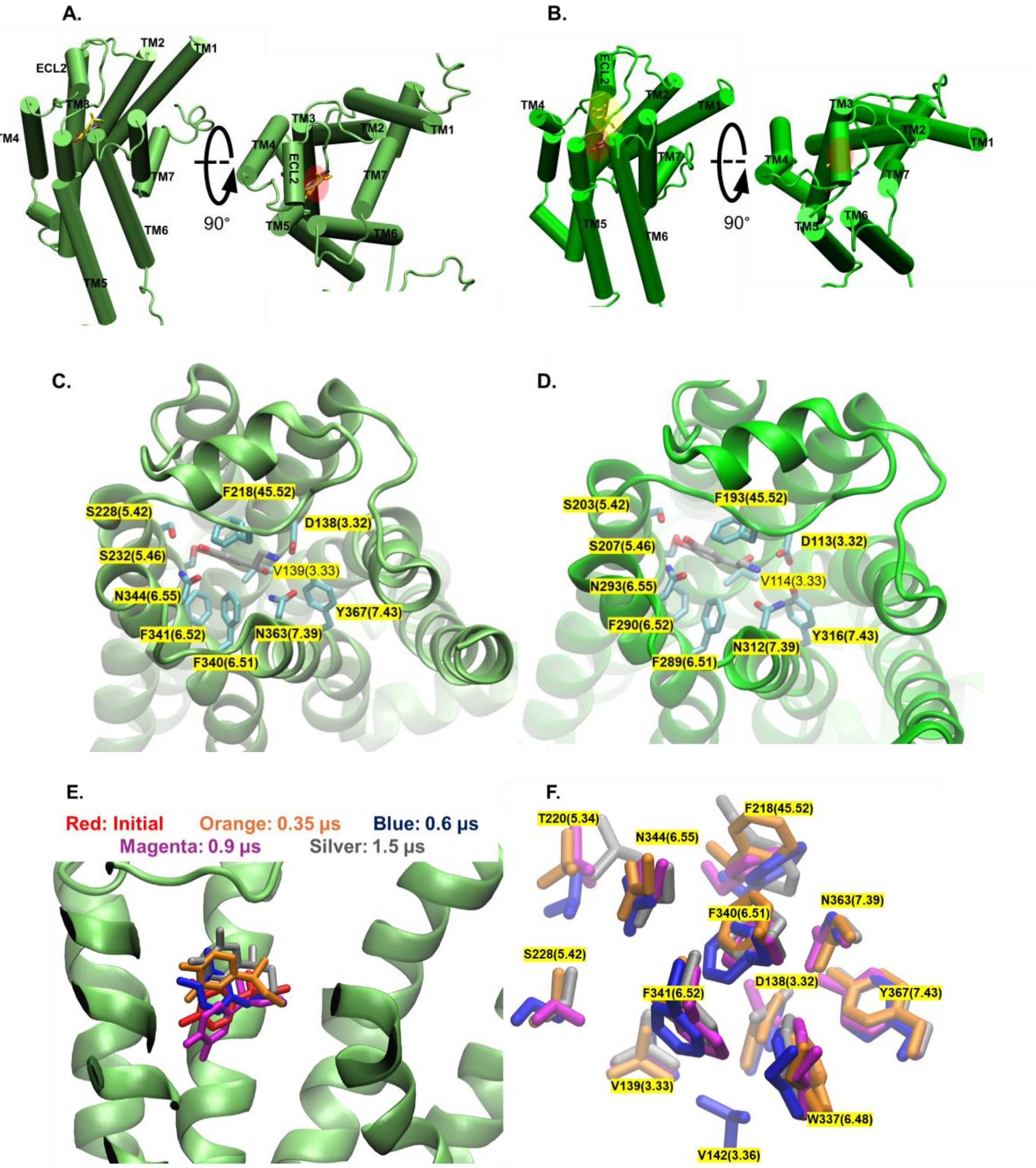

Figure S1. *Structural dynamics and interaction analysis of partially dissociated NE(+) in β_1AR and β_2AR systems via Anton 2 MD simulation. (A) Overview of the partially dissociated NE(+) in β_1AR. (B) Overview of the partially dissociated NE(+) in β_2AR. The receptor is shown by green ribbons, initial NE(+) position by red wireframe, whereas partially dissociated final NE(+) position is shown using yellow wireframe representation. (C) The initial pose of NE(+) and its main interactions with β_1AR. (D) The initial pose of NE(+) and its main interactions with β_2AR. (E) NE(+) poses, distinctly colored, captured at various points along the simulation trajectory for the β_1AR-G_s-GTP1 system. (F) The amino acid residues interact with NE(+) at various time points, color-coded to match the corresponding NE(+) pose. Amino acids residues were labeled with residue one-letter names, numbers and Ballesteros-Weinstein (BW) numbering scheme in parentheses (when available).*

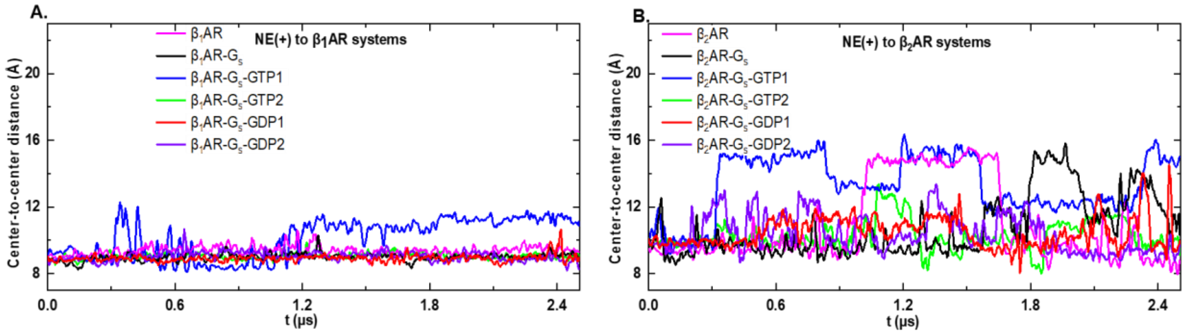

C.

```
CLUSTAL O(1.2.4) multiple sequence alignment

sp|P07550|ADRB2_HUMAN      ---------MGQPGNGSAF------------LLA----PNGSHAPDHDVTQERDEVWVVG 35
sp|P08588|ADRB1_HUMAN      MGAGVLVLGASEPGNLSSAAPLPDGAATAARLLVPASPPASLLPPASESPEPLSQQWTAG 60
                                     .:*** *:             **.    * .   *  :  :  .: *..*

sp|P07550|ADRB2_HUMAN      MGIVMSLIVLAIVFGNVLVITAIAKFERLQTVTNYFITSLACADLVMGLAVVPFGAAHIL 95
sp|P08588|ADRB1_HUMAN      MGLLMALIVLLIVAGNVLVIVAIAKTPRLQTLTNLFIMSLASADLVMGLLVVPFGATIVV 120
                           **::*:**** ** ******.****  ****:** ** ***.******* ******: ::

sp|P07550|ADRB2_HUMAN      MKMWTFGNFWCEFWTSIDVLCVTASIETLCVIAVDRYFAITSPFKYQSLLTKNKARVIIL 155
sp|P08588|ADRB1_HUMAN      WGRWEYGSFFCELWTSVDVLCVTASIETLCVIALDRYLAITSPFRYQSLLTRARARGLVC 180
                              * :*.*:**:***:****************:***:******:******: :** ::

sp|P07550|ADRB2_HUMAN      MVWIVSGLTSFLPIQMHWYRATHQEAINCYANETCCDFFTNQAYAIASSIVSFYVPLVIM 215
sp|P08588|ADRB1_HUMAN      TVWAISALVSFLPILMHWWRAESDEARRCYNDPKCCDFVTNRAYAIASSVVSFYVPLCIM 240
                            ** :*.*.***** ***:**  :** .** : .****.**:*******:******* **

sp|P07550|ADRB2_HUMAN      VFVYSRVFQEAKRQLQKIDKSEGRFHVQNLSQV--------------------------- 248
sp|P08588|ADRB1_HUMAN      AFVYLRVFREAQKQVKKIDSCERRFLGGPARPPSPSPSPVPAPAPPPGPPRPAAAAATAP 300
                           .*** ***:**::*::***..* **

sp|P07550|ADRB2_HUMAN      EQDGRTGHGLRRSSKFCLKEHKALKTLGIIMGTFTLCWLPFFIVNIVHVIQDNLIRKEVY 308
sp|P08588|ADRB1_HUMAN      LANGRAG-KRRPSRLVALREQKALKTLGIIMGVFTLCWLPFFLANVVKAFHRELVPDRLF 359
                             :**:*   * *  ..*:*:***********.*********:.*:*:.:: :*: ..::

sp|P07550|ADRB2_HUMAN      ILLNWIGYVNSGFNPLIYCRSPDFRIAFQELLCLRRSSLKAYGNGYSSNGNTGEQSGYHV 368
sp|P08588|ADRB1_HUMAN      VFFNWLGYANSAFNPIIYCRSPDFRKAFQGLLCCARRAARRRHA---THGDRPRASGCLA 416
                           :::**:**.**.***:********* *** ***  * : :       ::*:  . **  .

sp|P07550|ADRB2_HUMAN      EQ-----------EKENKLLCEDLP--GTEDFVGHQGTVPSDNIDSQGRNCSTND---SL 412
sp|P08588|ADRB1_HUMAN      RPGPPPSPGAASDDDDDDVVGATPPARLLEPWAGCNGGAAADSDSSLDEPCRPGFASESK 476
                           .            :.::.::    *    * :.* :* . :*. .* .. *  .    *

sp|P07550|ADRB2_HUMAN      L     413
sp|P08588|ADRB1_HUMAN      V     477
                           :
```

Figure S2. *Analysis of NE(+) partial dissociation and sequence alignment of β_1AR and β_2AR. (A) Time series of center-to-center distances (CCD) between NE(+) and β_1AR in β_1AR systems. (B) Time series of CCD between NE(+) and β_2AR in β_2AR systems. (C) Multiple sequence alignment for β_1AR and β_2AR. The ECL2 region is marked in red, while the ECL3 region is in orange.*

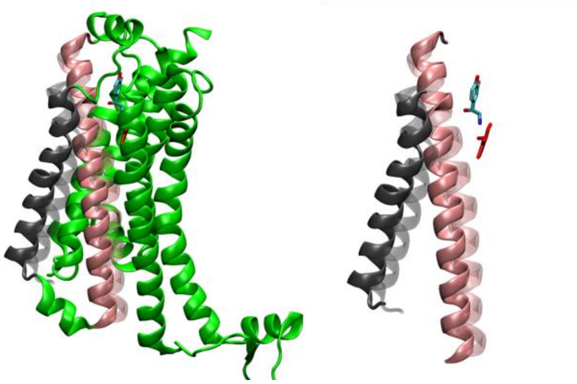

***Figure S3**. The conformation of TM3 (pink) and TM4 (black), when NE(+) in the β_2AR system shows highest level of partial dissociation at 1.2 μs of Anton 2 MD simulation (main Figures 2B and 2E). The transparent pink and black helices represent their initial conformations for comparison. The rest of the β_2AR is shown by green cartoons. The red molecule indicates the initial position of NE(+), while the cyan molecule indicates partially dissociated NE(+).The right panel shows only TM3 and TM4 for clarity.*

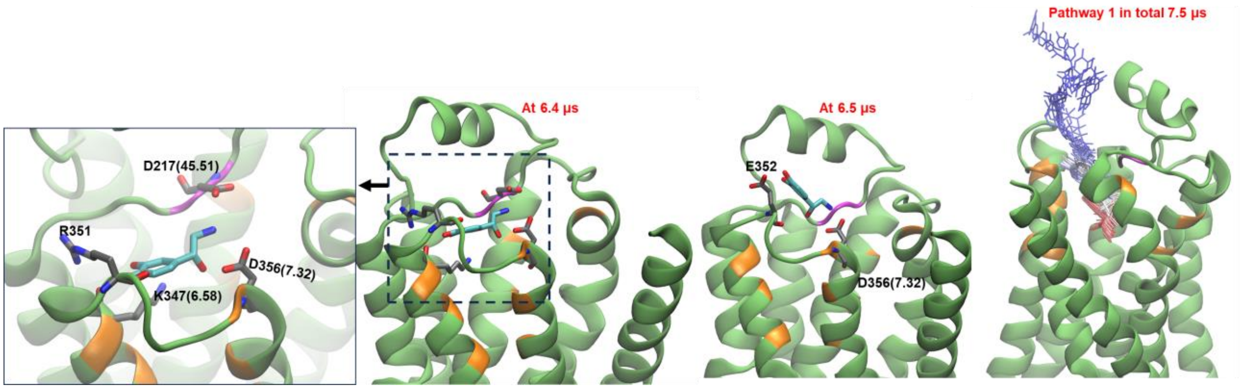

***Figure S4**. Complete dissociation pathway 1 of NE(+) in the β_1AR system (right panel) with snapshots captured at different simulation times (left and middle panels), based on the weighted ensemble (WE) run. In main-text Figure 5A, captured at 6.1 μs simulation time along pathway 1 from the β_1AR WE run, NE(+) became ensnared by a cage formed by ionic residues (D45.51, D7.32, K6.58) and aromatic residues (F45.52, F7.35, I2.64) located on TM2, TM6, TM7, and ECL2. Subsequently, NE(+) transitioned past the aromatic residues and interacted solely with the ionic residues, as depicted here in the left and middle panels captured at 6.4 μs and 6.5 μs simulation times, respectively, showing interaction with amino acid residues on TM6, TM7, and ECL2.*

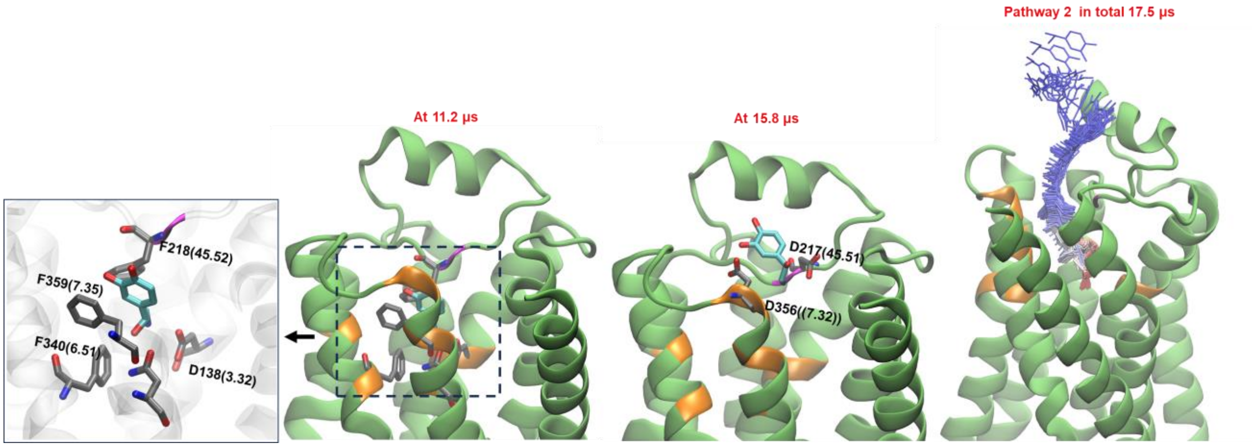

Figure S5. *Complete dissociation pathway 2 of NE(+) in the β_1AR system (right panel) with snapshots captured at different simulation times (left and middle panels), based on the weighted ensemble (WE) run. In main-text Figure 5B, captured at 7.3 μs simulation time along pathway 2 from the β_1AR WE run, NE(+) was ensnared by a cage comprising different ionic and polar residues (D3.32, N7.39, N6.55) and aromatic residues (F45.52, F6.51) located on TM3, TM6, TM7, and the inner side of ECL2. Subsequently, NE(+) transitioned past the aromatic residues and interacted solely with the ionic residues, as depicted here in the middle panel captured at 15.8 μs simulation time, showing interaction with amino acid residues on TM7 and the inner side of ECL2.*

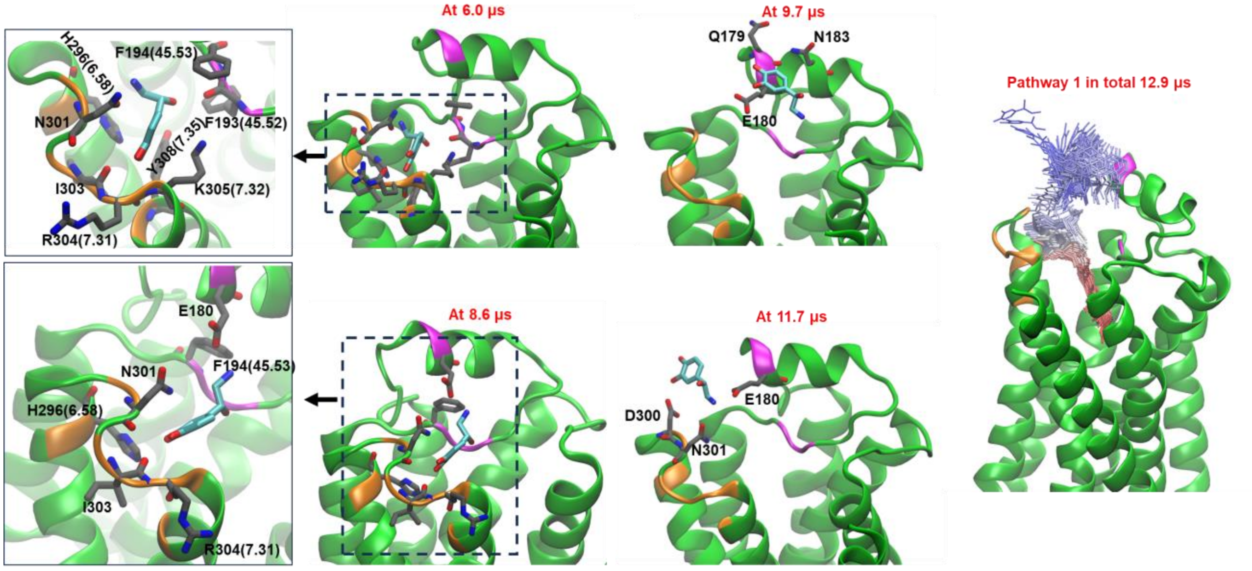

Figure S6. *Complete dissociation pathway 1 of NE(+) of the β_2AR system (right panel) with snapshots captured at different simulation times (left and middle panels), based on the weighted ensemble (WE) run. Main-text Figure 5C displays the snapshot captured at 8.6 μs simulation time along pathway 1 from the β_2AR WE run. At this point, NE(+) is ensnared by a cage formed by both aromatic and ionic residues on TM6, TM7, ECL3, and both inner and outer sides of ECL3. Following this, NE(+) alternates between ECL3 and ECL2, interacting solely with the ionic residues, as depicted here in the bottom middle panel at 11.7 μs simulation time.*

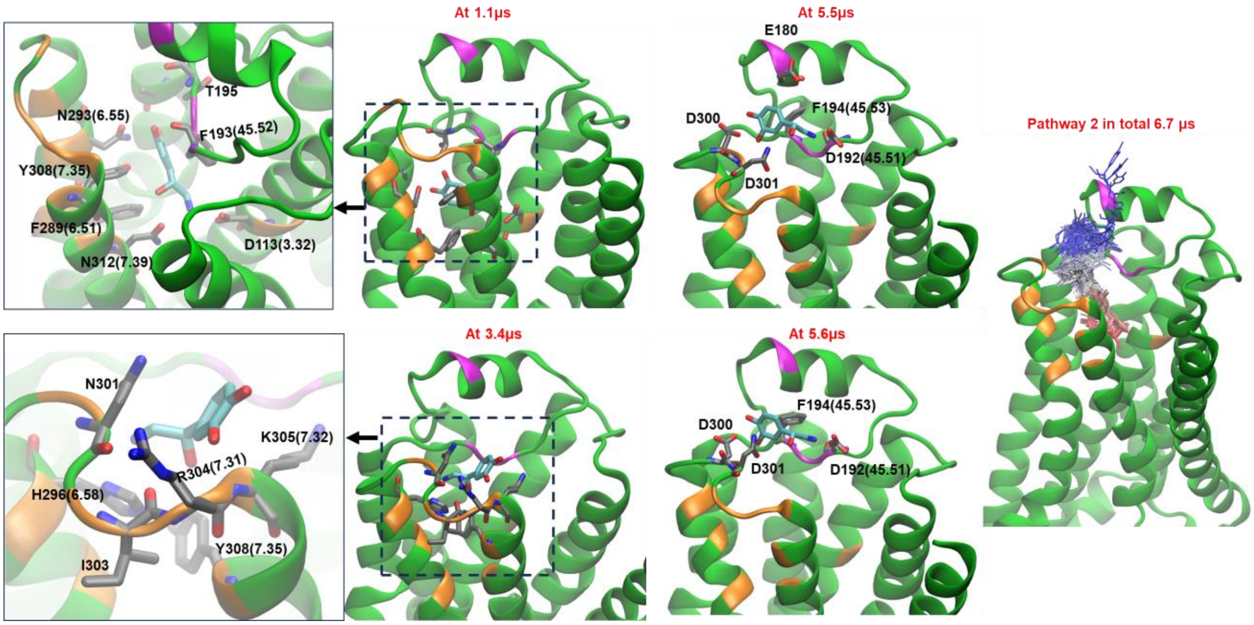

Figure S7. *Complete dissociation pathway 2 of NE(+) of the β$_1$AR system (right panel) with snapshots captured at different simulation times (left and middle panels), based on the weighted ensemble (WE) run. Main-text Figure 5D illustrates the snapshot taken at 3.3 μs simulation time along pathway 2 from the β$_2$AR WE run. At that point (3.3 μs), NE(+) interacts with both aromatic and ionic/polar residues, but exclusively on ECL3, TM6, and TM7 without involving ECL2. Subsequently, NE(+) transitions between the top of TM6 and the inner and outer sides of ECL2, as shown here in the middle panels at 5.5 and 5.6 μs simulation times.*

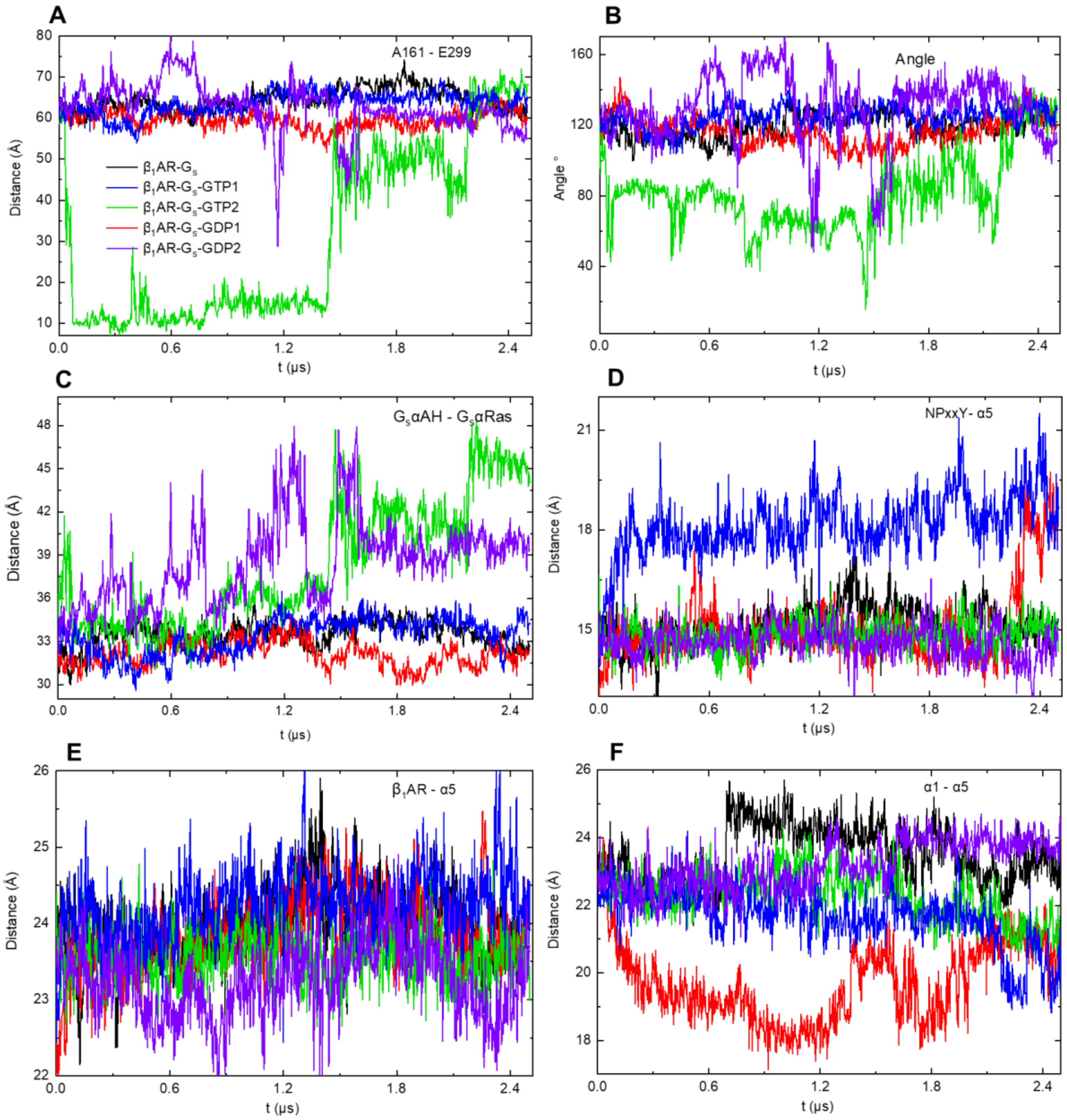

Figure S8*. Time series of geometric criteria from all-atom Anton 2 MD simulations of NE(+) bound β_1AR-G_s systems with and without GTP/GDP binding to G_s.* ***(A)*** *$G_s\alpha$ A161 to E299 distance indicating protein conformational changes (opening or closing). In the β_1AR-G_s-GDP2 system (panel A), $G_s\alpha$ initially became more open during the first 0.8 μs, followed by a transient transition to a closed state at around 1.2 μs before returning to an open state during the subsequent 1 μs simulation;* ***(B)*** *angle between two vectors in $G_s\alpha$AH and $G_s\alpha$Ras domains indicates the relative orientation of two domains. Vector 1 goes through $G_s\alpha$AH and its residue A161 centers, vector 2 goes through $G_s\alpha$Ras and its residue E299 centers;* ***(C)*** *distance between centers of $G_s\alpha$AH and $G_s\alpha$Ras domains;* ***(D)*** *distance between centers of NPxxY motif (on the TM7 of β_1AR) and $G_s\alpha$ α5 helix indicating protein-protein partial dissociation;* ***(E)*** *distance between centers of β_1AR and $G_s\alpha$ α5 indicating α5 dislocation / partial dissociation; (****F****) $G_s\alpha$ α1 to α5 center-to-center distance indicating relative movement of α1 and α5 helices. (Centers of geometry were used for the distance and angle measurements.)*

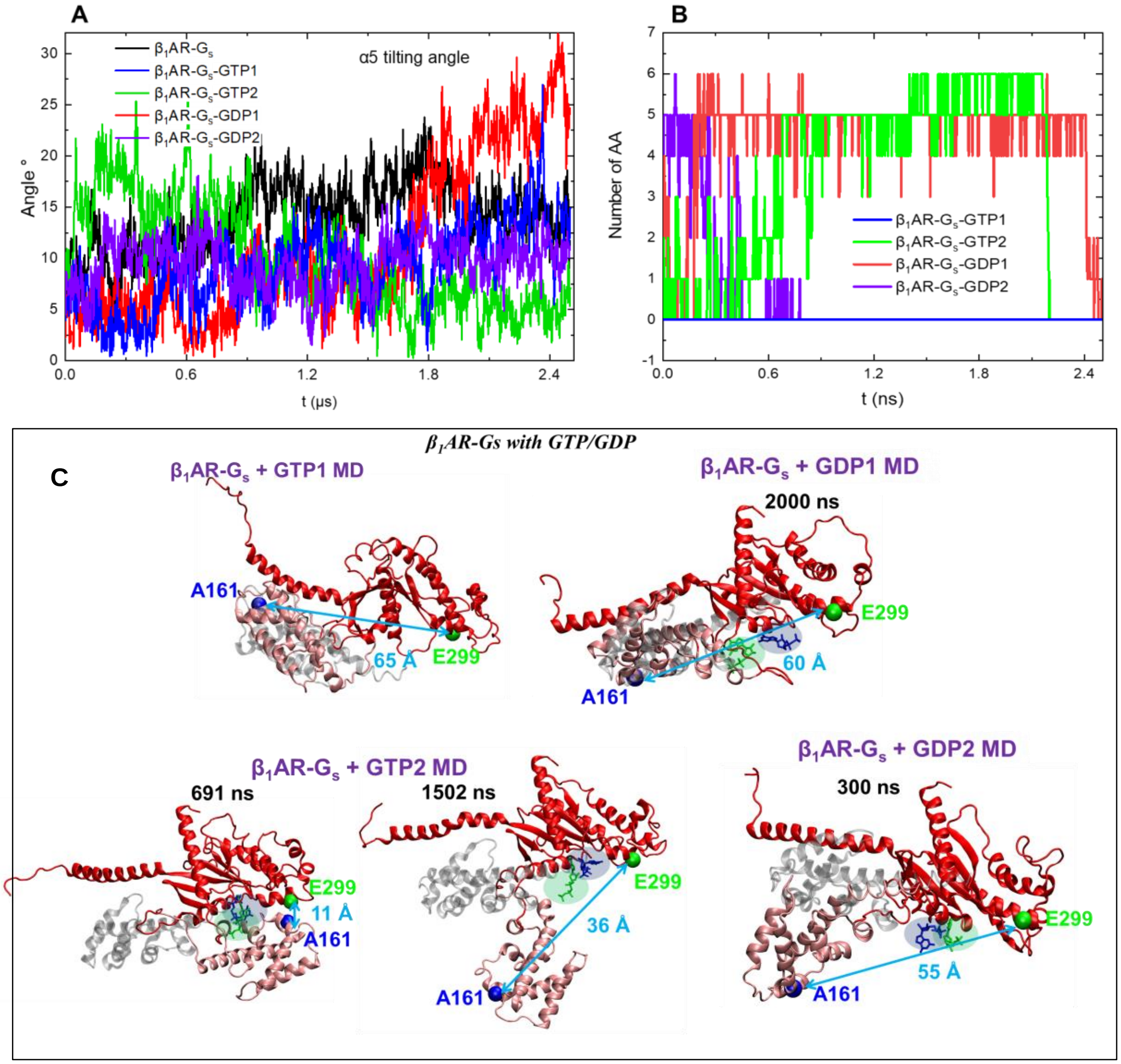

***Figure S9**. Time series of geometric criteria and GTP/GDP binding from all-atom Anton 2 MD simulations of NE(+) bound β_1AR-G$_s$ systems with and without GTP/GDP binding to G$_s$. **(A)** α5 tilting angle, the angle between the initial and final positions of α5 helix of G$_s$α; **(B)** the number of amino acid residues (AA) of G$_s$α within 3 Å of GTP/GDP indicating the binding and unbinding of GTP/GDP from G$_s$α. As depicted in panel B, the GDP binding at site 2 (GDP2) mostly occurred during the first 0.8 μs before dissociation from the G$_s$α. The G$_s$α in the β_1AR-G$_s$-GTP1 system was predominantly in an open state, and GTP initially docked at site 1 (GTP1) did not stay bound at all (panel B). For the β_1AR-G$_s$-GTP1, β_1AR-G$_s$-GTP2, and β_1AR-G$_s$-GDP2 systems, there were no significant changes in the α5 tilting angle when compared to the apo G$_s$ (panel A). However, while demonstrating less conformational changes, the G$_s$α in the β_1AR-G$_s$-GDP1 system showed an increase in the α5 tilting angle during 1.8-2.4 μs prior to GDP unbinding when compared to apo G$_s$. **(C)** Snapshots captured when GTP and GDP bind to G$_s$α. Green shaded molecules: initial positions of GTP/GDP; Blue shaded molecules: Final positions of GTP/GDP; Grey protein: initial position of α-helical domain of G$_s$α (G$_s$αAH); Pink protein: final position of G$_s$αAH; Red protein: Final position of Ras-like GTPase domain of G$_s$α (G$_s$αRas).*

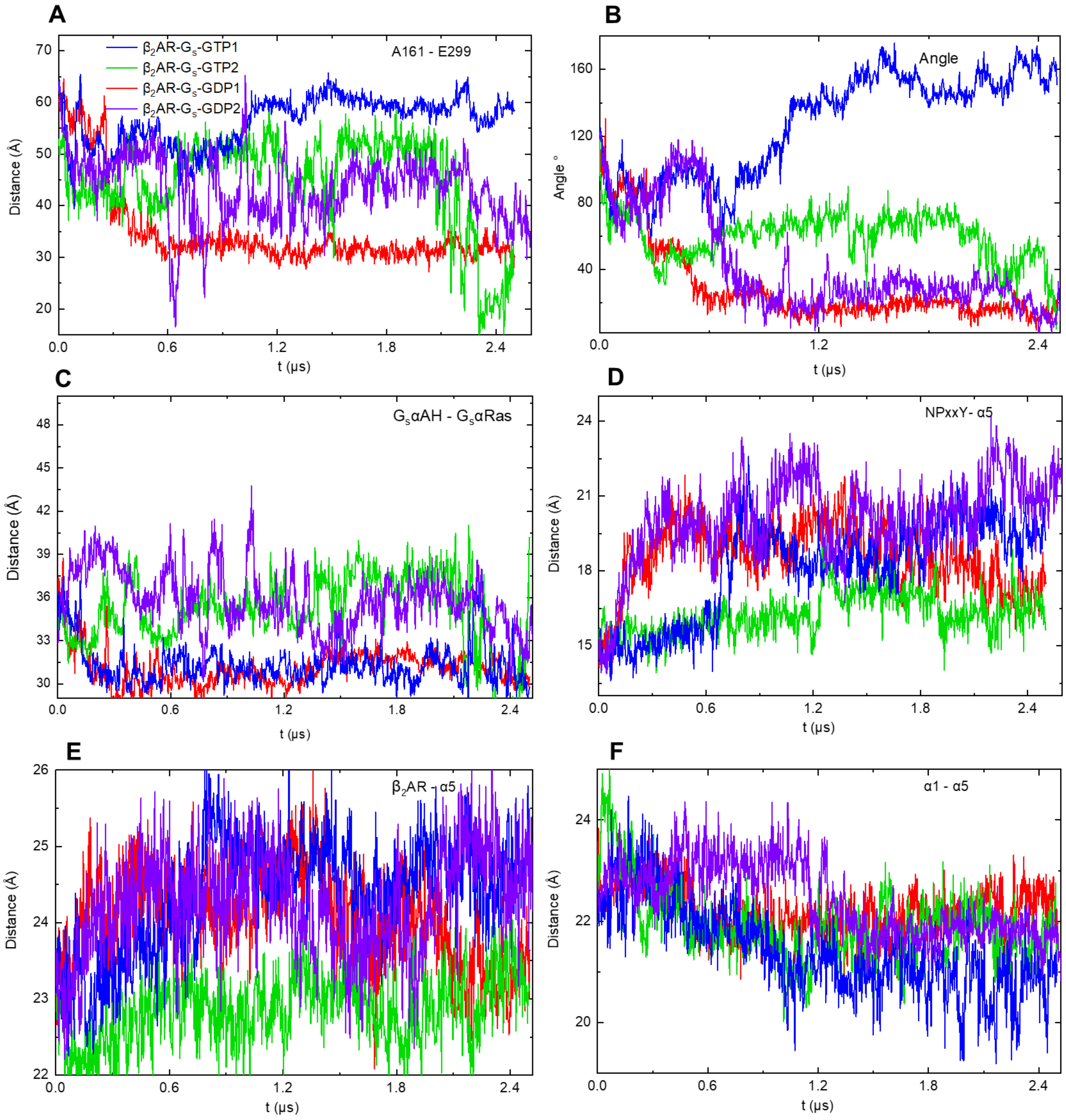

***Figure S10**. Time series of geometric criteria from all-atom Anton 2 MD simulations of NE(+) bound β_2AR-G_s systems with and without GTP/GDP binding. **(A)** $G_s\alpha$ A161 to E299 distance indicating protein conformational changes (opening or closing); **(B)** angle between two vectors in $G_s\alpha$AH and $G_s\alpha$Ras domains indicating the relative orientation of two domains. Vector 1 goes through $G_s\alpha$AH and residue A161 centers; vector 2 goes through $G_s\alpha$Ras and residue E299 centers; **(C)** distance between centers of $G_s\alpha$AH and $G_s\alpha$Ras domains; **(D)** distance between centers of NPxxY motif (on the TM7 of β_2AR) and $G_s\alpha$ α5 helix indicating protein-protein partial dissociation; **(E)** distance between centers of β_2AR and $G_s\alpha$ α5 indicating α5 dislocation / partial dissociation; **(F)** $G_s\alpha$ α1 to α5 center-to-center distance indicating relative movement of α1 and α5 helices. (The geometric centers were used for the distance and angle measurements.)*

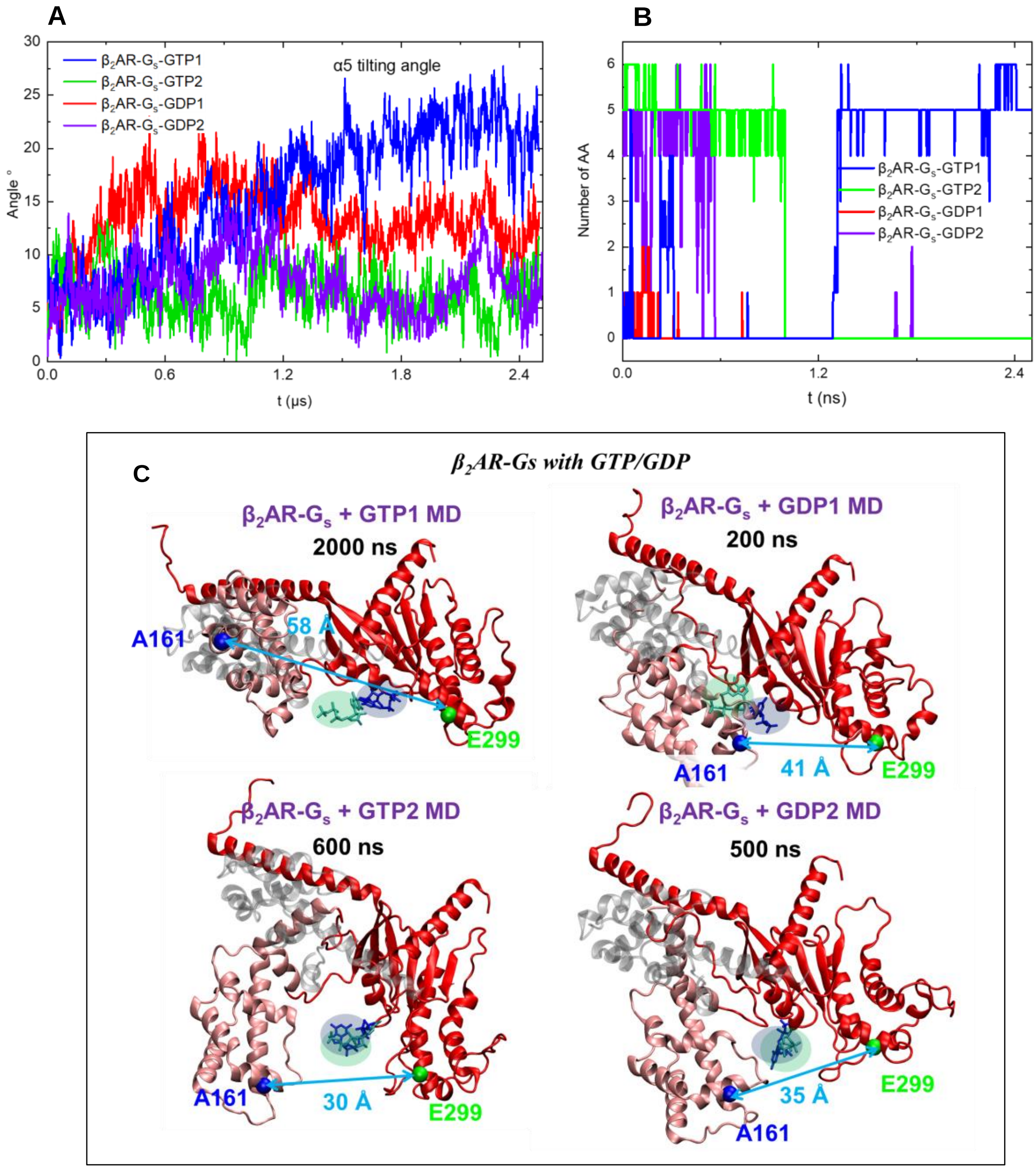

***Figure S11**. Time series of geometric criteria and GTP/GDP binding information from all-atom Anton 2 MD simulations of NE(+) bound β_2AR-G_s systems with and without GTP/GDP binding to G_s. **(A)** α5 tilting angle, the angle between the initial and final positions of α5 helix of $G_s\alpha$; **(B)** The number of amino acid residues (AA) of $G_s\alpha$ within 3 Å of GTP/GDP indicating binding and unbinding of GTP/GDP to/from $G_s\alpha$. **(C)** Snapshots captured when GTP and GDP bind to $G_s\alpha$. Green shaded molecules: initial positions of GTP/GDP; Blue shaded molecules: Final positions of GTP/GDP; Grey protein: initial position of α-helical domain of $G_s\alpha$ ($G_s\alpha$AH); Pink protein: final position of $G_s\alpha$AH; Red protein: Final position of Ras-like GTPase domain ($G_s\alpha$Ras).*

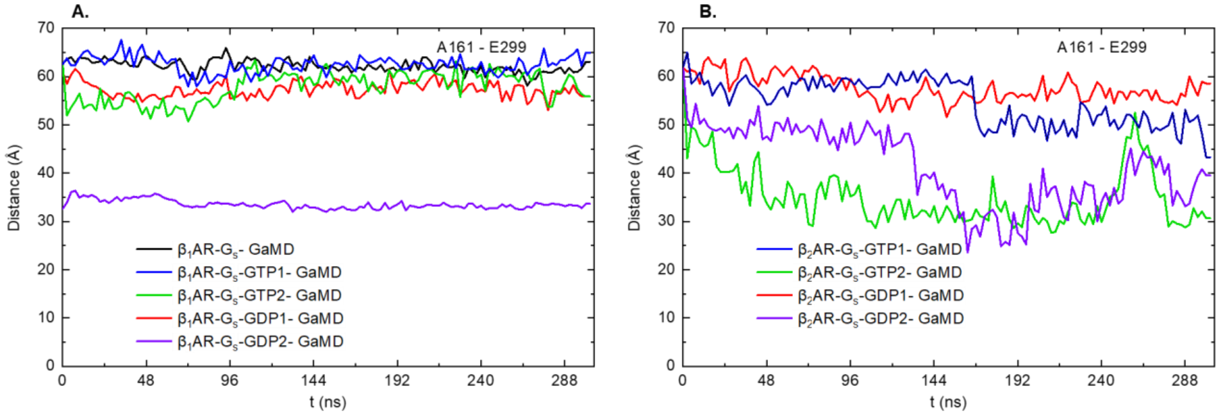

Figure S12. *Time series of $G_s\alpha$ A161 to E299 distance indicating protein conformational changes (opening or closing) from all-atom GaMD simulations.* ***(A)*** *for β_1AR-G_s systems and* ***(B)*** *for β_2AR-G_s systems.*

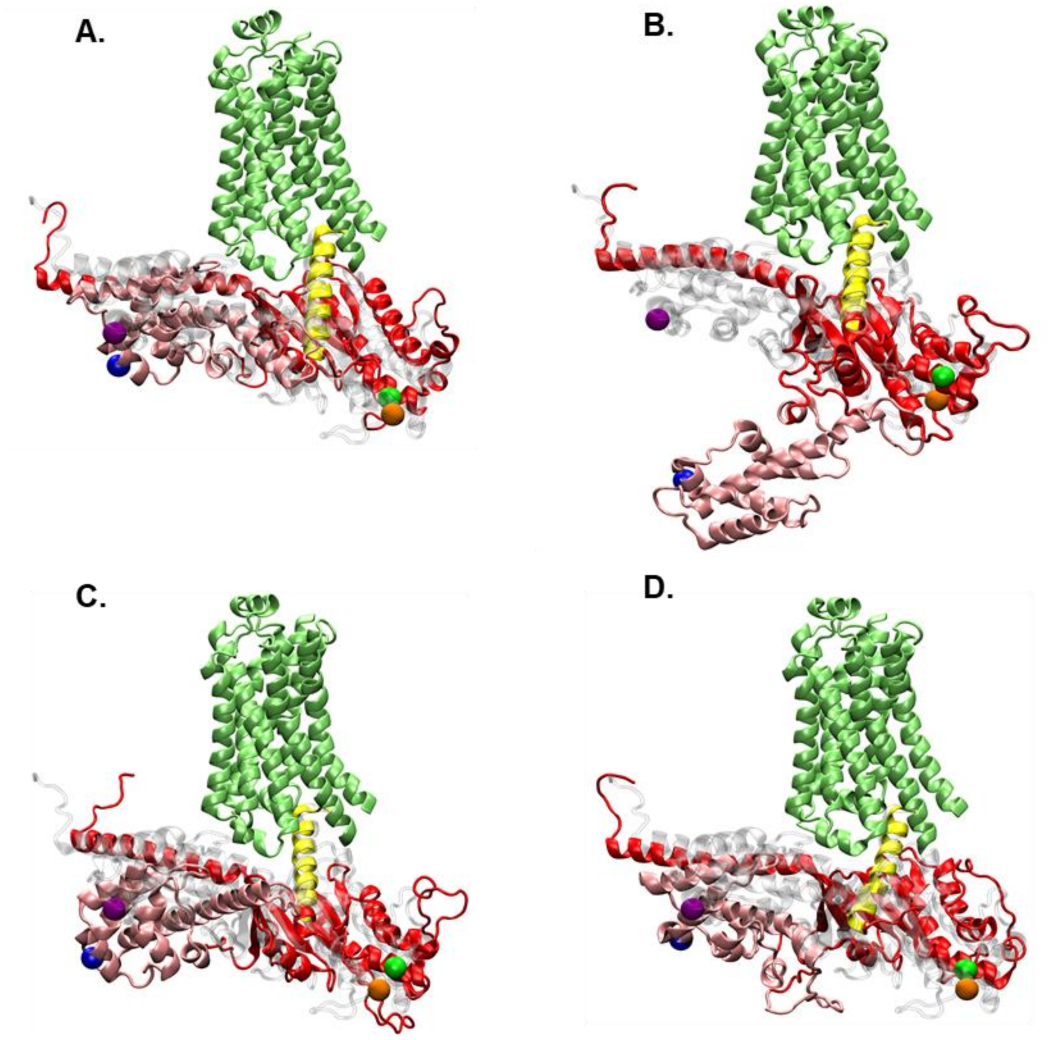

Figure S13. *Mutual selective conformations of β_1AR and $G_s\alpha$ in β_1AR-G_s-GDP systems with highlighted residue interactions and structural alignments.* ***(A-B)*** *Mutual selective conformations of β_1AR and $G_s\alpha$ from the β_1AR-G_s-GDP systems corresponding to the main Figure 8A-B.* ***(C-D)*** *The other mutual selective conformations of β_1AR and $G_s\alpha$ from the β_1AR-G_s-GDP systems. The proteins shown by grey traces are the initial poses of G_s based on their PDB structures, the intracellular loops 3 (ICL3) were omitted in the conformation clustering due to its flexible nature. Residues A161 on the $G_s\alpha$AH domain and E299 on the $G_s\alpha$Ras domain are shown as blue and green balls (corresponding to C_α atoms) in the mutual selective conformations, while they are shown in purple and orange, respectively, in the initial poses. Protein structures are aligned with respect to the receptor without the ICL3.*

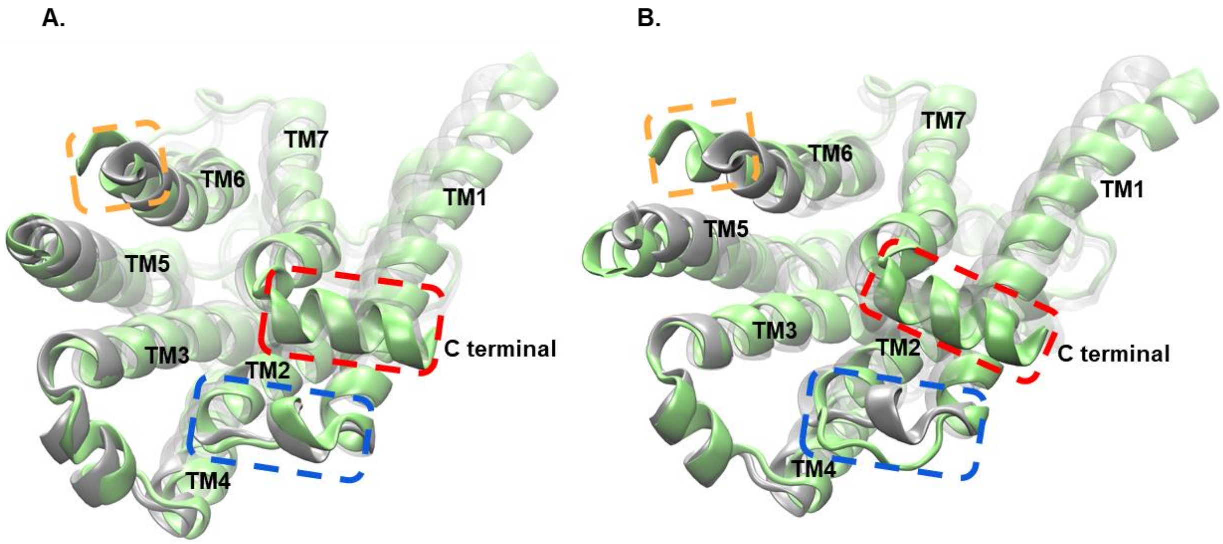

Figure S14. *The other representative conformations of β_1AR when they form mutual selective poses with G_s in the β_1AR-G_s-GDP systems corresponding to 2 different clusters* ***(panels A and B).*** *Proteins in green are the representative conformations. Proteins shown by grey traces are the initial poses of βARs based on their PDB structures. ICL3 and the last 7 amino acid residues in the C-termini were omitted in conformation clustering due to their flexible nature. The terminus of TM6 is shown in the orange dashed box; the truncated C terminus is marked by the red dashed box; the ICL1 in between TM1 and TM2 is shown in the blue dashed box.*

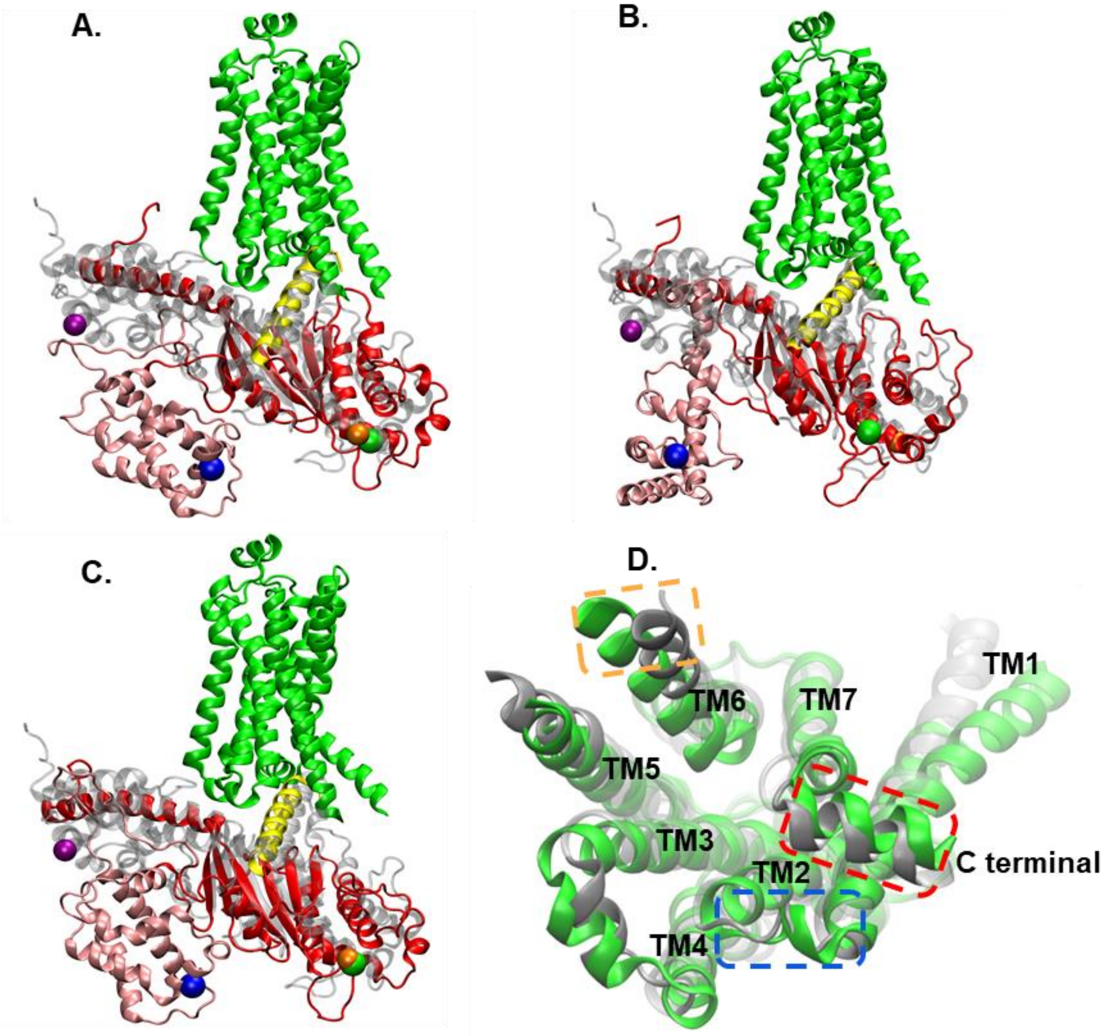

Figure S15. *The three mutual selective conformations/poses of β_2AR and $G_s\alpha$ captured from the clustering of conformations in the β_2AR-G_s-GDP systems. (A-B) Mutual selective conformations of β_2AR and $G_s\alpha$ from the β_2AR-G_s-GDP systems corresponding to the main Figure 8C-D. (C) The other mutual selective conformations of β_2AR and $G_s\alpha$ from the β_2AR-G_s-GDP systems. (D) The conformations of β_2AR when forming mutual selective poses with G_s in the β_2AR-G_s-GDP systems corresponding to the protein complex structure shown in panel C. The proteins in grey traces are the initial poses of βARs based on their PDB structures. The intracellular loops 3 (ICL3) were omitted in conformation clustering due to their flexible nature. Residues A161 on the $G_s\alpha$AH domain and E299 on the $G_s\alpha$Ras domain are shown as blue and green balls (corresponding to C_α atoms) in the mutual selective conformations, while they are shown in purple and orange, respectively, in the initial poses. ICL3 and the last 7 amino acid residues in the C-termini were omitted in conformation clustering due to their flexible nature. The terminus of TM6 is shown in the orange dashed box; the truncated C terminus is marked by the red dashed box; the ICL1 in between TM1 and TM2 is shown in the blue dashed box.*
